# A Social Inference Model of Idealization and Devaluation

**DOI:** 10.1037/rev0000430

**Published:** 2023-08-21

**Authors:** Giles W. Story, Ryan Smith, Michael Moutoussis, Isabel M. Berwian, Tobias Nolte, Edda Bilek, Jenifer Z. Siegel, Raymond J. Dolan

**Affiliations:** 1 Division of Psychiatry, University College London; 2 Max Planck-University College London Centre for Computational Psychiatry and Ageing Research, University College London; 3 Laureate Institute for Brain Research, Tulsa, Oklahoma, United States; 4 Wellcome Centre for Human Neuroimaging, University College London; 5 Princeton Neuroscience Institute, Princeton University; 6 Anna Freud National Centre for Children and Families, London, United Kingdom; 7 Mortimer B. Zuckerman Mind Brain Behavior Institute, Columbia University

**Keywords:** splitting, polarization, computational psychiatry, social inference, borderline personality disorder

## Abstract

People often form polarized beliefs, imbuing objects (e.g., themselves or others) with unambiguously positive or negative qualities. In clinical settings, this is referred to as dichotomous thinking or “splitting” and is a feature of several psychiatric disorders. Here, we introduce a Bayesian model of splitting that parameterizes a tendency to rigidly categorize objects as either entirely “Bad” or “Good,” rather than to flexibly learn dispositions along a continuous scale. Distinct from the previous descriptive theories, the model makes quantitative predictions about how dichotomous beliefs emerge and are updated in light of new information. Specifically, the model addresses how splitting is context-dependent, yet exhibits stability across time. A key model feature is that phases of devaluation and/or idealization are consolidated by rationally attributing counter-evidence to *external* factors. For example, when another person is idealized, their less-than-perfect behavior is attributed to unfavorable external circumstances. However, sufficient counter-evidence can trigger switches of polarity, producing bistable dynamics. We show that the model can be fitted to empirical data, to measure individual susceptibility to relational instability. For example, we find that a latent categorical belief that others are “Good” accounts for less changeable, and more certain, character impressions of benevolent as opposed to malevolent others among healthy participants. By comparison, character impressions made by participants with borderline personality disorder reveal significantly higher and more symmetric splitting. The generative framework proposed invites applications for modeling oscillatory relational and affective dynamics in psychotherapeutic contexts.

People sometimes form polarized beliefs about themselves and others. Thus, others can be *idealized*, and considered to have exceptionally positive qualities that they do not in reality possess, or *devalued*, and considered to have unrealistically negative qualities (Beck et al., 2015; Hinshelwood, 1989; Linehan, 1993). Polarized views of self or others feature in several mental health conditions and personality dispositions. For example, an oscillation between idealization and devaluation of others is a feature of borderline personality disorder (BPD; American Psychiatric Association, 2013; World Health Organization, 1992, 2018). By contrast, people with paranoid personality focus on extreme negative appraisals of others, manifested as a tendency to misconstrue the neutral or friendly actions of others as hostile, and to form “unsubstantiated ‘conspiratorial’ explanations of events” (World Health Organization, 1992). Similar dynamics, applied to the self, can feature as narcissistic personality traits, where “exaggerated self-appraisal may be inflated or deflated, or vacillate between extremes” (American Psychiatric Association, 2013).

Theories derived from psychoanalysis propose that idealization and devaluation imply polarization in a person’s internal model of self or others, referred to as *splitting* (Akhtar & Byrne, 1983; Fairbairn, 1952a; Kernberg, 1967, 1985; Klein, 1946; Kohut, 1966). A related, and more general, concept in cognitive psychology is *dichotomous thinking* (Arntz & Veen, 2001; Beck et al., 2015; Linehan, 1993; Napolitano & McKay, 2007; Veen & Arntz, 2000), which entails a tendency “to evaluate experiences in terms of mutually exclusive categories rather than to see experiences as falling along continua” (Veen & Arntz, 2000, p. 23). Here, we adopt the simpler term, splitting, although there is much overlap between the two concepts.

We introduce a social inference model of splitting, wherein accumulated observations can activate latent representations of others as either “all-good” or “all-bad.” The model captures a computational structure common to both cognitive and psychoanalytic theories, therefore bridging psychiatric and psychotherapeutic concepts across traditions. Before describing the model, we discuss existing theoretical and empirical research relevant to a splitting concept. We first discuss theories of splitting derived from various psychological perspectives. Second, we discuss relevant contemporary research on impression formation and causal attribution.

## Theories of Splitting

Theorists from various fields of psychology have proposed that dichotomous internal models of self and other are associated with unstable self-esteem and volatile relationships in adulthood (for overviews see Beck et al., 2015; Bender & Skodol, 2007; Crowell et al., 2009; Fonagy & Luyten, 2009; Kernberg, 1967; Kohut, 2013). As we review below, these theories converge on an idea that internal models of self and other are shaped by developmental experiences. On the one hand, attentive parenting is viewed as helping a developing child to acquire a contextualized and nuanced understanding of emotion, contributing to an emerging sense of self. On the other hand, both innate and environmental factors can impede emotional development, leading to a persistence of developmentally earlier, fragmented emotional representations.

### Object Relations Theory

Splitting gained prominence in psychoanalytic thinking with the development of the so-called *object-relations theory* (Gomez, 1997). Here, “object” is used in the sense of “subject” and “object” and refers to anything to which a person forms a relational attachment. Accordingly, this branch of psychoanalysis is concerned with how people represent their social relationships. In object-relations theory, splitting is classically seen as an early developmental mechanism by which an infant structures its experience by discriminating between positive and negative affect (Gomez, 1997; Hinshelwood, 1989; Klein, 1946; Zepf, 2012). In early development (the first year of life), an infant is said to lack a representation of self or other as enduring circumscribed entities, with a mixture of properties, and instead experiences disconnected states of extreme satisfaction and frustration (Hinshelwood, 1989; Klein, 1946; Steiner, 1992).

Splitting is also considered a psychological defense mechanism, serving to reduce anxiety by separating sources of security and threat, thereby preventing generalization of threat (Feldman, 1992; Hinshelwood, 1989; Kernberg, 1967, 1985; Klein, 1946; Kohut, 1966, 2013). This disposition is thought to be enhanced by defensively attributing feelings of aggression or frustration to others, who in turn come to be experienced as actively hostile (Feldman, 1992; Klein, 1946). Consequently, a relationship to another that is experienced as entirely hateful may be the basis of devaluation. Conversely, a child might also attribute feelings of love to others, who are experienced as entirely loving, an arrangement which forms the basis of idealization (Steiner, 1992). (In psychoanalytic terminology, external attribution of feelings that originate from the self is referred to as “projection”; Hinshelwood, 1989).

Object-relations theory describes how split representations mature through increasing integration as development proceeds. Fragmented aspects of caregiving experience are gradually brought together to form representations of self and other as whole entities, with a mixture of good and bad qualities (Steiner, 1992; Winnicott, 1945). Such integration is thought to help a child to manage frustration, and to form relationships. For example, representing a parent with a mixture of emotional qualities ensures that their bad aspects are buffered by an expectation of the good. This more integrated representation is associated with a stage of “object constancy,” wherein the child’s bond with its caregiver becomes a “stable and enduring inner relation independent of need-satisfaction” (Akhtar, 1994, p. 441; Freud, 1965; Hartmann, 1952; Mahler et al., 1975).

Psychoanalytic theorists have proposed that, to fully achieve object constancy, the child needs the help of its caregivers buffer extreme affective shifts (Gergely & Watson, 1996; Mahler et al., 1975; Winnicott, 1967; for a review see Akhtar, 1994). Accordingly, the degree of integration achieved during development is said to vary between individuals (Fairbairn, 1952b). Furthermore, object-relations theories propose that, although integration often supersedes splitting, developmentally earlier modes of relating remain latent, and can come to the fore under conditions of stress (Bion, 1963; Fairbairn, 1943; Steiner, 1987, 1992; Winnicott, 1965).

#### Borderline Personality Organization

Building on object-relations theories, Kernberg influentially suggested that split images of self and other are central to the pathology of BPD. Kernberg proposed that “borderline personality organization” derives from heightened aggressive impulses, which are either innate, or secondary to excessive frustration during development, and which threaten to annihilate more benign internal images of the self and others (Kernberg, 1967, 1985, 2015). Profound splitting is putatively necessary to preserve a feeling of security in the face of these internal threats, by keeping apart good and bad aspects of self and others (Kernberg, 1967). Thus, according to Kernberg, a polarized and fragmented view of others as either “all-good” or “all-bad,” a remnant of incomplete integration during development, leads to unstable relationships in adulthood, which are characterized by shifts between idealization and devaluation. Horowitz (2004) elaborates a similar idea from the perspective of interpersonal psychology, proposing that, due to inconsistent or abusive parenting, people with BPD have difficulty integrating good and bad attributes of their early caregivers, and as a consequence in later life they are prone to vacillate between assessments of people close to them as either “all-good” or “all-bad.”

### Studies of Emotion Concept Learning

Psychoanalytic theories, such as those described above, are derived inductively from clinical material and draw on metaphorical constructs (such as “ego” or “part objects”) whose biological and behavioral referents are not defined (see Target & Fonagy, 1996; Zepf, 2012). Furthermore, accounts of splitting in infant development are difficult to verify experimentally, owing to the challenges of accessing early infantile emotional experiences. However, empirical studies of how older children acquire emotion concepts suggest a broadly similar trajectory (Barrett, 2006; Hoemann et al., 2020). Thus, preschool children first discriminate positive from negative affect, before learning to differentiate emotions of the same valence, such as anger, sadness or fear (Widen & Russell, 2003, 2008). Furthermore, children progress from describing emotion in all-or-nothing terms (e.g., as a friend’s behavior making them completely angry, sad, or jealous), to recognizing grades and mixtures of emotion (Harter & Buddin, 1987; Harter & Whitesell, 1989; Westen, 1991; Whitesell & Harter, 1989). Taken together, object-relations accounts and studies of emotional concept acquisition both suggest that a developing child first recognizes a coarse separation between positive and negative affect, consistent with “splitting.” While object-relations theory focuses on the subsequent integration of these “bad” and “good” aspects of self and others, studies of emotional development also describe how a child acquires an increasingly differentiated and graded set of emotional concepts. Both accounts describe a child’s increasing capacity to represent mixtures of emotion associated with a single object.

### Cognitive and Mentalization-Based Theories

Cognitive theories similarly emphasize how dichotomous reasoning, shaped by traumatic early life experience, can underpin beliefs about self and others in adulthood (Beck et al., 2001, 2015; Pretzer & Beck, 1996). In cognitive behavioral therapy (CBT), the relational and affective instability that characterize BPD are seen as arising from maladaptive patterns of thoughts, feelings and memories regarding the self and others, referred to as “schemata” (Young et al., 2003), which can be expressed as all-or-nothing logical statements. For example, a person with BPD might believe “If I trust someone I’ll be abused or abandoned” (Beck et al., 2015). When such beliefs are activated, people with BPD are prone to view others in extreme and excessively simplistic ways (Arntz et al., 1999; Bhar et al., 2008; Butler et al., 2002; Del Pozo et al., 2018; Geiger et al., 2014). Thus, according to the cognitive theory, abrupt switches between idealized or devalued views of others can result from activation of underlying schemata with a dichotomous structure.

Linehan’s biosocial theory (Crowell et al., 2009; Linehan, 1993) also places affective dysregulation and associated dichotomous reasoning at the center of borderline psychopathology. Linehan (1993) proposed that BPD develops within an invalidating childhood environment, wherein a child’s emotional expression is neither tolerated nor understood. As a result, the child does not learn to tolerate or understand their *own* emotional responses, and therefore tends to experience extremes of emotion. Dialectical behavior therapy (DBT), a treatment model for BPD constructed around biosocial theory, emphasizes rebalancing such polarities of emotion and thought (Dimeff & Koerner, 2007; Lynch et al., 2006).

A more recent therapeutic approach situates dichotomous reasoning in BPD within a broader deficit in a capacity to understand and interpret mental states, referred to as “mentalizing” (Allen & Fonagy, 2006; A. Bateman & Fonagy, 2013; Fonagy & Bateman, 2008; Nolte et al., 2019). Here, extreme or simplistic appraisals of others, termed “hypomentalizing,” are seen as resulting from heightened affect in relational situations (Agrawal et al., 2004; A. Bateman & Fonagy, 2013; Choi-Kain et al., 2009; Fonagy & Bateman, 2008; Lyons-Ruth et al., 2005; Nolte et al., 2019). Conversely, mentalizing is seen as promoting affect regulation, by providing a context in which to appraise affect (Nolte et al., 2019). Drawing on psychodynamic theory, Fonagy and colleagues describe how a responsive parent processes the child’s emotional needs, communicating these back to the child in a digestible form; through this process of “contingent mirroring” the parent helps the child to identify its own emotional states and their causes, laying the foundations for mentalizing (Fonagy, 2002; Fonagy & Target, 1996; Gergely & Watson, 1996; Target & Fonagy, 1996; Winnicott, 1967).

In support of these ideas, there is evidence that adults diagnosed with personality disorders make dichotomous evaluations of others’ emotions in interpersonal settings (Arntz & Veen, 2001; Bender & Skodol, 2007; Kramer et al., 2013; Moritz et al., 2011; Napolitano & McKay, 2007; Perry et al., 2013; Preißler et al., 2010; Roepke et al., 2013; Sieswerda et al., 2005; Veen & Arntz, 2000; Zanarini et al., 2009). In a notable study, healthy adults, adults with BPD, and adults with other forms of personality disorder were asked to evaluate emotions displayed by different characters in film clips (Veen & Arntz, 2000). Consistent with dichotomous thinking, people with BPD were found to make significantly more extreme emotional evaluations than both sets of controls. Other studies find that dichotomous thinking, rated using a clinical questionnaire, correlates with overall symptom severity in BPD (Moritz et al., 2011) and occurs in the evaluation of positive as well as negative content (Napolitano & McKay, 2007).

## Splitting as Distorted Causal Inference

The aforementioned clinical theories describe splitting as an over-simplification and/or distortion in how people interpret the emotions or motives underlying others’ behavior (see, Bender & Skodol, 2007). However, previous approaches have not elaborated in quantitative terms how such distorted causal reasoning might give rise to relational dynamics seen in personality disorders. Key unanswered questions in this regard are as follows: (a) Why do phases of idealization and devaluation exhibit stability across time? (b) Why can such phases undergo sharp changes of polarity? and (c) Why is dichotomous thinking context-dependent? A model accounting for these effects would naturally lend itself to measuring individual susceptibility to relational and affective instability and enhance a functional understanding of these phenomena.

Here, we address these questions with the framework of probabilistic inference. To do so, we formalize idealization and devaluation as *causal hypotheses* about others’ mental states that are of extreme valence and firmly held. Pertinent to this account is extensive research examining how people discern the causes of others’ behavior (Ajzen & Fishbein, 1975; Ajzen & Holmes, 1976; Hastie, 1984; Heider, 1958; Hilton & Slugoski, 1986; Jones & Davis, 1965; Kelley, 1967, 1973; Kelley & Michela, 1980; Malle, 2011; Ybarra, 2002). As we discuss below, such studies offer a putative explanation for why split beliefs can persist despite often being inaccurate.

### Causal Attribution and Impression Formation

A comprehensive body of research has analyzed when and how adults attribute behavior to causes either *internal* or *external* to the actor, respectively, termed “dispositional” and “situational” causes. Such studies support an idea that dispositional attributions are favored when behavior appears diagnostic of a person’s idiosyncratic character (e.g., Ajzen & Holmes, 1976; Ferguson et al., 2019; Heider, 1958; Jones & Davis, 1965; Kelley, 1967; Ybarra, 2002). For example, if a person displays consistent behavior across time, this is found to support dispositional attributions (for a review see Kelley & Michela, 1980), particularly when exhibited across different situations (Himmelfarb, 1972), and when such behavior eschews social or occupational conventions (Jones et al., 1961; Thibaut & Riecken, 1955).

The other side of the coin, as Kelley and Michela (1980) describe, is that “out-of-character” behavior provokes *situational* attributions (Bell et al., 1976; Crocker et al., 1983; Frieze & Weiner, 1971; Hayden & Mischel, 1976; Karaz & Perlman, 1975; Kulik, 1983). For example, a liked person’s good behavior and a disliked person’s bad behavior both elicit dispositional attributions, whereas inconsistent behavior elicits situational attributions (Regan et al., 1974). Thus, a liked person’s good behavior is often attributed to their good character, while their bad behavior is assigned to situational factors (e.g., stress or intoxication). By contrast, a disliked person’s bad behavior is often attributed to their malign character, and their good behavior to situational factors (e.g., societal norms or an ulterior motive). In other words, once people have formed an impression of another’s disposition or ability, impression-congruent behavior tends to be attributed to dispositional factors, while impression-incongruent behavior is attributed to situational factors.

#### Motivated Versus Procedurally Rational Attribution

As Kim et al. (2020) outline, two broad classes of processing might underlie effects of initial impressions on subsequent information processing: motivated cognition and procedural rationality. Explanations based on motivated cognition propose that people are inclined to believe in their own good-fortunes (Eil & Rao, 2011; Hughes & Zaki, 2015; Huys et al., 2012; Kunda, 1990; M. E. P. Seligman, 1991; Seligman & Schulman, 1986; Zuckerman, 1979). In support of this idea, people exhibit a range of self-serving biases (for a review see Bromberg-Martin & Sharot, 2020), and often minimize responsibility for their offensive behavior by blaming others or external circumstances (Maruna & Mann, 2006). However, a motivated cognition theory cannot easily explain why external-situational attributions are often symmetric with respect to valence, reversing direction depending on expectations (Regan et al., 1974). For example, when people expect to perform *badly* on a task, they tend to attribute their *successes* to external factors (e.g., luck) and their failures to internal factors (e.g., a lack of ability; Bradley, 1978; Feather, 1969; Feather & Simon, 1971a, 1971b). A similar self-devaluing attributional style is observed in depression (Forgas et al., 1990; M. E. Seligman et al., 1979). Such findings are not readily explained in terms of a self-serving bias.

An alternative view is that countervailing Information is “explained away” to situational factors in a procedurally rational manner (see Gershman, 2019; Kim et al., 2020). Procedural rationality implies that people arrive at conclusions following a reasoning process that is consistent, given their prior expectations and knowledge (Simon, 1978). For instance, if a person experiences frequent success and becomes confident in their own ability, it is plausible for them to attribute failure to unusual, situational factors for the simple reason that they rarely fail.

In support of a procedurally rational account, prior expectations influence subsequent information processing across contexts. For instance, in attributions of task performance, unexpected outcomes (whether successes or failures) are attributed more to luck than to ability (Jones et al., 1968; for reviews, see Ajzen & Fishbein, 1975; Zuckerman, 1979). In addition, numerous studies show that peoples’ interpretations of political events are skewed toward their existing partisan convictions (for reviews, see Gerber & Green, 1999; Jern et al., 2014; Katz & Feldman, 1962; Sigelman & Sigelman, 1984; van Baar & FeldmanHall, 2022). As discussed by Kim et al. (2020), the more certain (or precise) is one’s prior impression, the more plausible it is that inconsistencies are due to supervening external causes (see also, Erdmann & Mathys, 2022). Gershman (2019) provides a detailed account of this process, with reference to how arbitrary “auxiliary hypotheses” can be invoked to explain away contradictions to an existing belief.

#### Situational Attribution Stabilizes Splitting

Here, we model how procedurally rational attribution to fictive situational causes can stabilize and consolidate idealization and/or devaluation across time. To do so, we formalize idealization and devaluation as resulting from activation of excessively certain and extreme representations of others’ dispositions. When such split representations are active, it is procedurally rational for a subject to attribute countervailing information to situational factors. The model proposed here has affinities with existing approaches to polarization of political beliefs. For example, using Bayesian latent variable models, Cook and Lewandowsky (2016) and Botvinik-Nezer et al. (2021) illustrate that rational attribution processes can account for an otherwise puzzling finding that people sometimes update their beliefs in diverging directions in response to the same information (Kuhn & Lao, 1996; Lewandowsky et al., 2012; Lord et al., 1979).

A novel feature of the current model is that polarized beliefs exist in a form of dynamic equilibrium with each other, and with more integrated representations. This arrangement has three key implications. First, by analogy with emotional development, the model allows the balance of polarized and integrated representations to be updated through experience. Second, polarization can reemerge from a background of more integrated functioning if observations become more extreme. Third, when an extreme belief is upheld, an opposite extreme belief is sometimes represented in latent form. Extreme beliefs can therefore abruptly switch polarity if sufficient counterevidence is observed.

## A Social Inference Model of Splitting

We formalize splitting within a probabilistic model wherein subjects infer dispositional (internal) and situational (external) causes of another person’s behavior. Our model follows myriad theories proposing that our brain evaluates probabilistic hypotheses about the hidden causes of its inputs by approximating Bayesian inference (Chater & Oaksford, 2008; Dunsmoor et al., 2015; Friston et al., 2016, 2017; Gershman, 2017; Gershman et al., 2013, 2015; Gershman & Blei, 2012; Gershman & Niv, 2012; Glimcher, 2004; Noorani & Carpenter, 2016; Tomov et al., 2018), which have previously been applied to social inference (e.g., Ajzen & Fishbein, 1975; Barnby et al., 2020; Diaconescu et al., 2020; Moutoussis, Fearon, et al., 2014; Moutoussis, Trujillo-Barreto, et al., 2014; Redcay & Schilbach, 2019; Reiter et al., 2019; Wellstein et al., 2020). Formally, Bayes’ theorem states how new information can be optimally combined with the prior knowledge to update belief in particular hypothesis as follows:

$$p(s|o)=\frac{p\left(s\right)p(o|s)}{p\left(o\right)}.$$ 1

Here, p(s|o) is the *posterior probability* of an hypothesis that the world is in state, *s*, given some observations, *o*. A *prior probability*, *p*(*s*) denotes level of belief in an hypothesis before seeing the data. A *likelihood* term, p(o|s) denotes the probability of observing such data, if the world were indeed in state *s*. The denominator, *p*(*o*) is a normalizing constant, which ensures the probabilities of alternative hypotheses sum to one.

The numerator of Bayes’ theorem describes how hidden states give rise to observations, and is therefore referred to as a *generative model*. In psychological terms, this corresponds to an internal model of how the world works. Inference entails working backward to discover which hidden (or latent) states best explain the available observations (Griffiths & Yuille, 2008, p. 19).

### Social Inference: An Example Scenario

To illustrate how Bayes’ rule can be applied to infer others’ intentions, consider the following scenario. You are due to meet a friend for coffee and they do not arrive on time. As you wait for your friend to arrive, you consider the possibility that they may not want to see you. However, you also consider alternative possibilities, including that they were simply delayed due to unforeseen signaling problems on the metro system.

**Figure 1 fig1:**
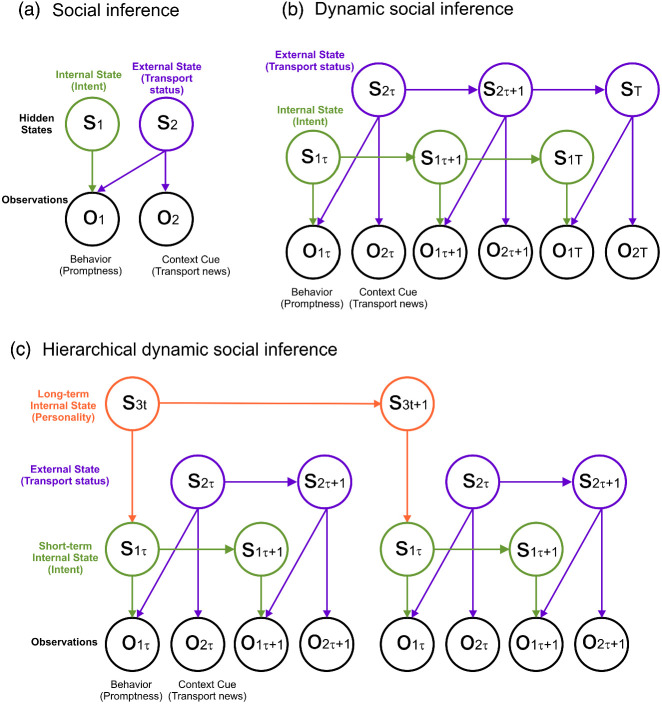
Models of Social Inference *Note*. (a) Probabilistic graph illustrating how hidden states generate observed variables. Here, another person’s behavior (*o*_1_, e.g., promptness in attending an appointment) depends on both their internal state (*s*_1_, e.g., motivation to attend) and an external state (*s*_2_, e.g., transport delays). A cue (*o*_2_, e.g., transport news) provides information about the external context (*s*_2_). (b) HMM specifying a propensity for hidden states to change over time. (c) A higher order (person state) representation, *s*_3_, is updated over a longer timescale, and entails a “dispositional” prior over internal state (e.g., personality), and a “situational” prior over external state (e.g., a person’s usual circumstances). Here, we consider an HMM with a single higher order timestep per trial, such that *s*_3_ is updated through trial-to-trial learning. HMM = hidden Markov model. See the online article for the color version of this figure.

Each possibility can be expressed as a random variable: the friend’s promptness in arriving (*o*_1_), the transport news (*o*_2_), their motivation to see you (*s*_1_) and metro delays (*s*_2_). These are depicted as nodes in a *directed graphical model* in Figure 1a. Here, promptness and transport news are observable variables, while reliability and metro delays are *hidden states*. In this example, promptness (*o*_1_) depends on both an intention to be on time (*s*_1_) and metro delays (*s*_2_). Transport news (*o*_1_) by contrast depends only on metro delays (*s*_2_). We can therefore write the joint distribution over observations and hidden states (i.e., how likely is any combination of observations and their hidden causes) as follows:

$$P(o_{1},\;o_{2},\;s_{1},\;s_{2})=P(o_{1}|s_{1},\;s_{2})P(o_{2}|s_{2})P(s_{1})P(s_{2}).$$ 2

[Fig fig1]

Belief in different hidden states can be updated in light of observations according to the Bayes’ rule as above.

The above arrangement can be further elaborated to express a time-dependence of hidden states, creating a hidden Markov model (HMM; Bishop, 2006; Chater & Oaksford, 2008). Consider, for example, that you meet your friend for coffee every morning, and that their promptness in arriving depends on a combination of their motivational state that morning and the status of the transport network. Here, rather than a graphical model, relationships between hidden states are described in a *state transition diagram*. A state transition diagram for an HMM based on the example above is shown in Figure 1b. Such a model specifies, in a *transition matrix*, a propensity for hidden states to change over time; for instance, the model specifies a probability that, if there were transport delays yesterday, there will also be transport delays today.

As shown in Figure 1c, beliefs can be updated across nested timescales. For instance, one might infer not only a friend’s intention each day, but also their prevailing intentions from 1 week to the next, corresponding to an assessment of their disposition or personality. This higher order (“person”) representation, *s*_3_, enters the model through a prior over starting states at the level below, $p\left(s_{1},s_{2}|s_{3}\right)$; in other words, an expectation about the other’s internal and external states (Figure 1c).

An hierarchical organization also features in contemporary computational models of human structure learning (Gershman, 2017; Gershman et al., 2015; Gershman & Niv, 2012; McCormack et al., 2015; Tenenbaum & Griffiths, 2001; Tomov et al., 2018), which have found application in modeling emotion concept learning (Smith et al., 2018; Smith, Parr, et al., 2019; Smith, Schwartenbeck, et al., 2020). Hierarchical structure is also in keeping with previous non-Bayesian models of intentional attribution. For example, Jones and Davis (1965) proposed that intentions are the data for inferring dispositions. Similarly, Trope’s (1986) two-stage model of dispositional inference posits that behavioral data are initially assigned intentional categories (e.g., “A’s reaction is hostile”), which in turn are used to deduce more enduring dispositions (e.g., “A is a hostile person”). We note here that Bayesian models are essentially models of commonsense reasoning. Furthermore, as pointed out by Bowers and Davis (2012), translating descriptive models into Bayesian language does not necessarily add explanatory power. However, posing descriptive theories in generative (Bayesian) terms allows inference to be simulated in complex, dynamical contexts, where commonsense predictions may not be intuitive.

### A “Split Hidden Markov Model”

We model social inference based on the above example, by equipping a simulated subject with an hierarchical generative model of the form shown in Figure 1c. An overview of this “split hidden Markov model” (split-HMM) is provided below and is summarized in Table 1 (see supplemental material, for technical model specifications). Under the model, a subject infers *internal* and *external* states, *s*_1_ and *s*_2_, respectively, corresponding to another person’s intention and their environmental context. These jointly generate two observations: the other person’s behavior, *o*_1_, and a contextual cue, *o*_2_. Both hidden states and observations are expressed as discrete quantities. We operationalize observed behavior along an eleven point ordinal scale from 0 to 1, representing the objective level of cost or benefit for the subject.[Table tbl1]

#### First-Level State Representation

**Figure 2 fig2:**
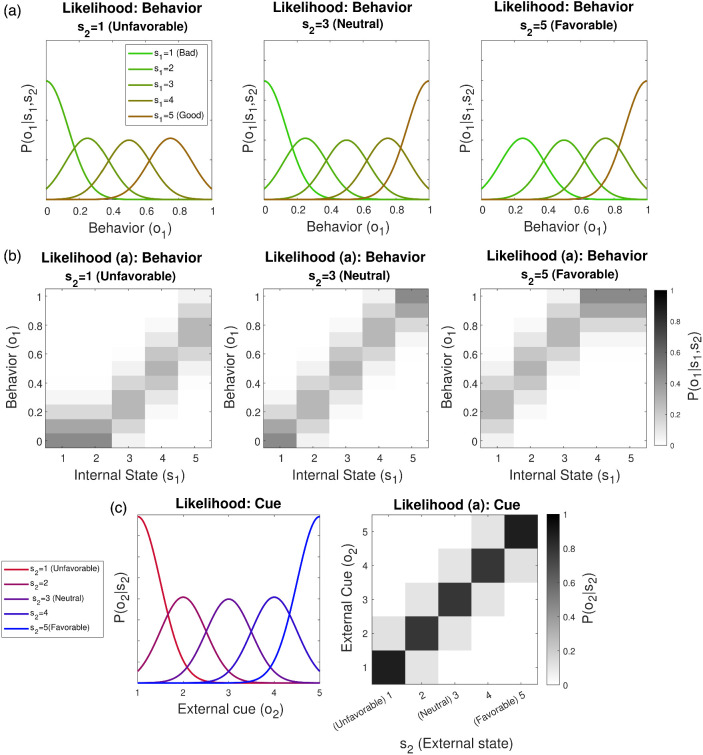
First-Level Model Specification *Note. Rows (a) and (b):* Likelihood distributions illustrating how internal (*s*_1_) and external hidden states (*s*_2_) jointly generate observed behavior, *o*_1_, displayed both as smoothed probability distributions (a), and as the discrete matrices used in the model (b). In (a) likelihood distributions are shown for each internal state (“intention”), such that better intentions tend to generate better behavior. An *Unfavorable* external state worsens behavior: For each internal state, the mode of the likelihood distribution is shifted toward poorer behavior (reaching a floor at *s*_1_ = 2). A *Favorable* external state improves behavior: For each internal state, the likelihood distribution is shifted toward better behavior (reaching a ceiling at *s*_1_ = 4). Here π_*o*1_ = 0.75. *Row (c):* Likelihood distributions illustrating how external states generate cues, *o*_2_, as smoothed distributions (left) and as a discrete matrix (right). As explained in the main text, we consider either informative (π_*o*2_ = 2, shown here) or uninformative (π_*o*2_ = 0.001) cues. Note that likelihood distributions are truncated and therefore more peaked at the extremes; this feature does not of itself generate splitting, which instead results from dispositional priors at the second level. See the online article for the color version of this figure.

At the first level of the model, a subject is equipped with a graded representation of internal states (*s*_1_), corresponding to varying degrees of benevolence in another’s intentions: *s*_1_ = {*Bad*, *Moderately Bad*, *Neutral*, *Moderately Good*, *Good*}. These five internal states map onto behavior in a realistic way, accounting for intermediate observations, according to a likelihood distribution, $P\left(o_{1}|s_{1}\right)$ (Figure 2a). The subject is also equipped with an external state dimension, *s*_2_, representing degrees of favorability in circumstances: *s*_2_ = {*Very Unfavorable*, *Unfavorable*, *Neutral*, *Favorable*, *Very Favorable*}. Here, unfavorable external circumstances worsen behavior (*o*_1_; which could occur despite Good intentions), while favorable circumstances improve behavior (which could occur despite Bad intentions). This is shown in Figure 2b. External factors can also generate contextual cues (*o*_2_; Figure 2c). A parameter, π_*o*1_, denotes the likelihood precision for behavior $P\left(o_{1}|s_{1},s_{2}\right)$. Higher values of π_*o*1_ entail a more deterministic mapping from internal states to behavior; this renders inference more sensitive to changes in the behavior observed in others. An equivalent parameter, π_*o*2_, denotes the likelihood precision for cues $P\left(o_{2}|s_{2}\right)$.[Fig fig2]

#### Second-Level State Representation

At the second level of the model, the subject represents longer timescale expectations about the other person and their circumstances. Here, *person* states (*s*_3_) generate prior expectations, $P\left(s_{1},s_{2}|s_{3}\right)$, about others’ internal and external states. Prior expectations over *internal* states, $P\left(s_{1}|s_{3}\right)$, encode a person’s disposition, or personality, while expectations over *external* states, $P\left(s_{2}|s_{3}\right)$, encode a person’s prevailing situation in the outside world (Figure 1c). We refer to these as “dispositional” and “situational” priors, respectively. A subject represents a mixture over three person states, *s*_3_ = {*Bad*, *Integrated*, *Good*}, which entail different dispositional and situational priors.

##### Dispositional Priors

**Figure 3 fig3:**
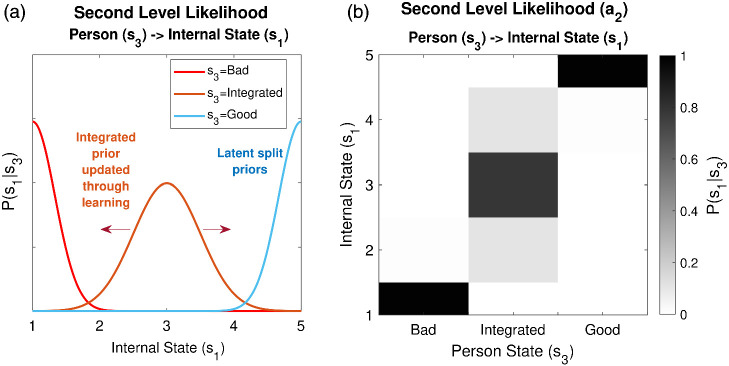
Second-Level Model Specification *Note*. Dispositional priors at the second level, illustrating how person states generate first-level internal states. These are displayed both as smoothed probability distributions (a), and as the discrete matrices used in the model (b). An integrated dispositional prior is updated through learning. The integrated prior displayed here has precision π_*s*1_ = 2. By contrast, split dispositional priors encode beliefs that others are either extremely Bad or extremely Good, formalized as two extreme unimodal priors, whose shapes are not modifiable through learning. See the online article for the color version of this figure.

As shown in Figure 3, we consider two forms of dispositional prior, $P\left(s_{1}|s_{3}\right)$. First, an *Integrated* unimodal prior, $P\left(s_{1}|s_{3}=Integrated\right)$, privileges a modal internal state, but also allows for others, in proportion to their relatedness to the modal state. We configure $P\left(s_{1}|s_{3}=Integrated\right)$ such that others’ intentions are expected to start as *Neutral* with some uncertainty around this estimate. This arrangement can be seen as arising out of healthy emotional development, whereby a differentiated representation of emotion (*s*_1_), is balanced by an integrated superordinate representation of a person as a whole (*s*_3_). Second, a subject can be equipped with *split* priors over dispositions, $P\left(s_{1}|s_{3}=Bad\right)$ and $P\left(s_{1}|s_{3}=Good\right)$, which prescribe a strong dominance of either *Bad* or *Good* internal states, respectively (we use capitalized italics to denote latent states of the model). Split priors could be hypothesized as arising from fragmented, developmentally earlier, models of others.[Fig fig3]

A parameter, π_*s*1_, determines the extent to which integrated dispositions constrain internal states at the level below. Higher values of π_*s*1_ prescribe a narrower range of internal states, rendering internal state inference *less* sensitive to changes in observed behavior. By contrast, the precision of split priors is fixed to a high value (π = 3).

##### Situational Priors

Situational priors, $P\left(s_{2}|s_{3}\right)$, are also conditioned on person states (*s*_3_), allowing expectations regarding external circumstances to potentially differ for different people or personalities. We initialize situational priors with a free parameter, ϕ_*Ext*_, bounded 0–1, which controls the weighting on nonneutral relative to neutral external states. Setting ψ_*Ext*_ = 0 denotes an expectation that *s*_2_ = {*Neutral*} for all *s*_3_. Changes in observed behavior are therefore attributed to changes in internal state. By contrast, increasing values of ψ_*Ext*_ encourage attribution to unfavorable or favorable external conditions. For *s*_3_ = *Bad*, when ψ_*Ext*_ > 0, neutral or favorable external attributions are possible, embodying an expectation that bad people can behave well due to ulterior motives or situational pressures. For *s*_3_ = *Good*, when ψ_*Ext*_ > 0, neutral or unfavorable external attributions are possible, embodying an expectation that good people can behave poorly in adverse contexts. For *s*_3_ = *Integrated*, we assume an equal prior weighting across all external states.

##### Person Priors

At the highest level of the model hierarchy, a Dirichlet distribution over person states, *P*(*s*_3_), is initialized with concentration parameters δ_*Bad*_, δ_*Integrated*_, and δ_*Good*_, where δ denotes the concentration parameter for each person representation. We constrain these parameters such that:

$$\delta_{Bad}=\psi_{Bad}\times \psi_{Split},\delta_{Integrated}=1-\psi_{Split},\delta_{Good}=(1-\psi_{Bad})\times \psi_{Split}.$$ 3

Thus, ψ_*Split*_ and ψ_*Bad*_ are subject-specific parameters, bounded between 0 and 1, controlling the prior degree of splitting and its asymmetry. Setting ψ_*Split*_ = 0, ensures *P*(*s*_3_ = *Integrated*) = 1, and thereby prevents splitting, while setting ψ_*Split*_ = 1 allows only splitting. Similarly, setting ψ_*Bad*_ = 0 ensures *P*(*s*_3_ = *Bad*) = 0, and thereby turns off a *Bad* mode, while setting ψ_*Bad*_ = 1 turns off a *Good* mode. Intermediate settings of ψ_*Split*_ and ψ_*Bad*_ allow a dynamic mixture between splitting and integration, and between *Bad* and *Good* modes.

#### Inference and Learning

*Inference* consists of inverting the model to estimate the hidden states likely to have generated observations. Model inversion is accomplished using a biologically plausible, variational method for approximating Bayesian inference (the details of which have been described elsewhere; Friston et al., 2017; for a tutorial review, see Smith et al., 2022). In brief, this method approximates posterior inference using a variational (marginal) message-passing algorithm that balances the predictive accuracy and complexity of posterior beliefs (Parr et al., 2019). Unlike the most common uses of this modeling framework, our model does not include policies for action.

Conditional relationships between states can also be learned across time. In the scheme proposed here, *learning* entails accruing information about which states a person is likely to occupy (formally, via accumulation of concentration parameters in Dirichlet priors). Thus, an agent first infers hidden states given their current model and observations, and subsequently, updates a generative model through learning. All learning takes place at the second level. We refer to a sequence of observations at the first level as a *trial*, after which the *second-level* model parameters are updated to mediate learning. The reader is referred to supplemental material for the update equations mediating learning. Table 1 summarizes which variables in the model are updated through learning.

#### Updating Dispositional and Situational Priors

An *Integrated* dispositional prior is updated by accumulating information about the relative frequency of different internal states (*s*_1_) at the level below, $P\left(s_{1}|s_{3}=Integrated\right)$. Thus, a subject with an *Integrated* prior is capable of inferring local changes in others’ mental states, while also learning about their overall disposition. The rate of belief updating is governed by the relative precision of priors and likelihoods at each level. For example, a precise dispositional prior means that inferred internal states change little in response to new observations, and trial-to-trial changes in the prior through learning are accordingly slower. Corresponding situational priors are also learned across time. By distinction, *Bad* and *Good* priors over dispositions are rigid. That is, we assume that the form of split priors, $P\left(s_{1}|s_{3}=Bad\right)$ and $P\left(s_{1}|s_{3}=Good\right)$, cannot change through learning (i.e., they remain extreme and precise).

#### Updating Person Priors: Splitting Versus Integration

Finally, the frequency of inferred person states, *s*_3_, is also learned across time, by updating a person prior. Thus, a subject can accumulate experience of how often *Bad*, *Good*, or *Integrated* representations best accounted for their observations. For example, by observing that others behave in a moderate and predictable manner, a subject might learn to represent others in an integrated fashion (i.e., accumulating a high prior probability of *s*_3_ = {*Integrated*}), a process analogous to healthy emotional development. Importantly, since such learning leaves the form of split priors unmodified, splitting can be reinstated, for example, in response to the unexpectedly extreme observations (cf. Dunsmoor et al., 2015; Gershman & Niv, 2012). This resembles an idea in psychodynamic theory that early developmental splitting exists “in equilibrium” with more integrated functioning and can reemerge under conditions of stress (Bion, 1963; Fairbairn, 1943; Steiner, 1987, 1992; Winnicott, 1965).

### Data and Code Availability

Computer code used for simulations is available on request to the authors.

## Simulation Results

We first show how a split-HMM can generate dynamics of idealization and devaluation seen in clinical settings, by simulating learning and inference under the split-HMM, when provided with changes in another person’s observed behavior over time. In each case, we generate observations from a prespecified series of internal and external states at the first level. For each epoch (i.e., timestep within a trial), the subject first observes a cue, *o*_2_, which gives information regarding the external state (information about transport delays), and subsequently observes the other’s behavior, *o*_1_ (degree of promptness). We simulate responses from 48 trials, with two first-level epochs per trial.

### Integrated Social Inference

**Figure 4 fig4:**
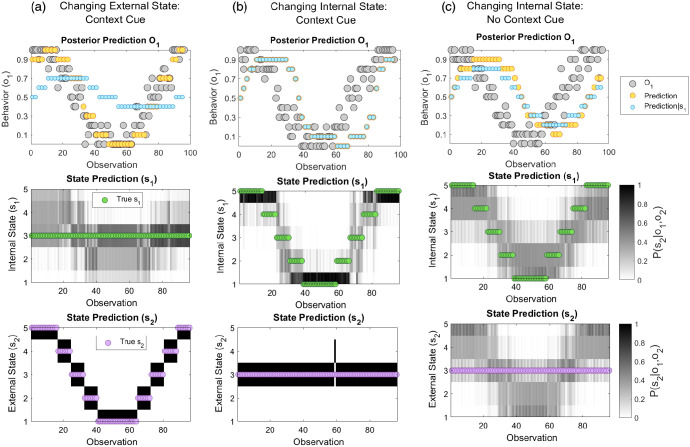
Inference With an Integrated Dispositional Prior *Note*. Simulated inference with an integrated dispositional prior (π_*s*1_ = 0.5). *Column (a)*: the subject observes both another’s behavior (o_1_, gray circles; π_*o*1_ = 0.25), and a cue (*o*_2_, not shown) reliably indicating external state changes (π_*o*2_ = 2). *Top panel*: observed behavior gradually worsens, before improving again. The subject accurately predicts changes in behavior. Predictions about upcoming behavior, after observing the cue, are shown as yellow circles. Predictions conditioned on internal state (assuming *s*_2_ = *Neutral*) are shown as blue circles. *Middle panel:* True internal states (*s*_1_, green circles) remain constant. The subject’s posterior predictions over hidden states, after observing both cue and behavior, are shown as grayscale shading. *Bottom panel:* The subject accurately tracks external state changes (*s*_2_, violet circles). *Column (b):* Changing behavior arises from changes in internal state. A cue (*o*_2_) reliably indicates (π_*o*2_ = 2) that external state does not change. *Column (c):* Without a reliable external state cue (π_*o*2_ = 0.001), the subject partly attributes internal state changes to external factors. See the online article for the color version of this figure.

To illustrate inference in the absence of splitting, we instantiate a broad, unimodal (*Integrated*) dispositional prior over internal states (ψ_*Split*_ = 0, π_*s*1_ = 0.5, π_*o*1_ = 0.25, ψ_*Ext*_ = 0.6). Figure 4 shows predicted behavior and expected hidden states after each observation. (The evolution of priors from trial-to-trial is shown in supplemental Figure S1). Here, the simulated subject smoothly tracks changes in the other’s behavior across time.[Fig fig4]

If the simulated subject is provided with reliable information about external states, in the form of a contextual cue (π_*o*2_ = 2), they accurately infer that changes in behavior are due to changes in *external* rather than internal states (Figure 4a). To appreciate this, consider the “meeting for coffee” scenario. After observing the friend’s lateness (*o*_1_), the propositions that they do not want to attend or that there are delays on the transport system both become more likely; however, after seeing the status of the metro system (*o*_2_) and finding that there are indeed delays, the subject is no longer certain of their friend’s bad intent. That is, evidence in support of this is *explained away* by the transport delays. As a result, beliefs about the other’s internal state remain relatively constant, despite changing behavior. Conversely, if context cues indicate that external state has *not* changed, the subject accurately attributes behavior to changes in internal state (Figure 4b).

We next reduce the precision of the likelihood mapping between external states and cues (π_*o*2_ = 0.001), so that cues are uninformative. As shown in Figure 4c, the subject is still capable of tracking the other’s behavior. However, they are unable to discern whether behavior results from internal or external factors. As a result, the subject makes attribution errors, attributing external state changes to internal factors and *vice versa*. Such inference is erroneous with respect to the ground-truth, but Bayes’ optimal given available information. This instantiates a previously proposed notion that attribution errors might arise from incomplete contextual information (Miller & Ross, 1975), and that arbitrary inference is greater in ambiguous scenarios (Beck, 1963).

Notably, in the absence of contextual cues (or where changing behavior is attributable to changing internal state), a subject’s predictions lag behind observed behavior (upper panels of Figure 4c and 4b). This arises since beliefs must be gradually accumulated from trial-to-trial via learning (e.g., accruing knowledge that a person is generally unmotivated to be on time). By contrast, where the external context is changing, precise contextual cues allow the subject to make accurate, prospective predictions of behavior (e.g., using transport news to predict that a person will be late; upper panel of Figure 4a).

### Devaluation Following Inference of a Bad Dispositional Prior

To illustrate devaluation, we introduce a latent *Bad* person state to the above model. To do so, we set the prior probability of a *Good* person to zero (ψ_*Bad*_ = 1) and configure *s*_3_ with increasing prior probabilities of *s*_3_ = {*Bad*}, achieved by increasing ψ_*Split*_. Here, context cues are uninformative (π_*o*2_ = 0.001) and other parameters are configured as for previous simulations (ψ_*Ext*_ = 0.6, π_*s*1_ = 0.5, π_*o*1_ = 0.25).

**Figure 5 fig5:**
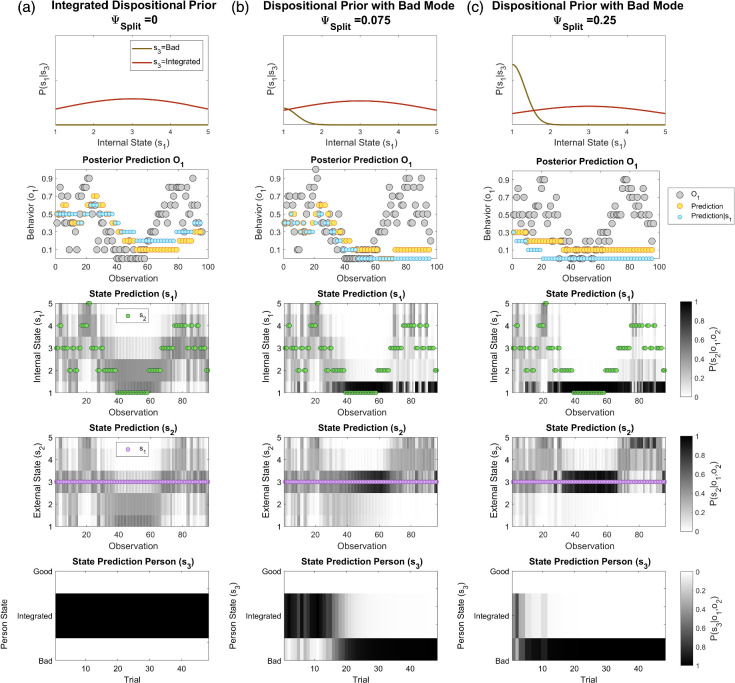
Inference With a Latent “Bad” Dispositional Prior: Devaluation *Note*. Simulated data are plotted as in Figure 4 (ϕ_*Bad*_ = 1, π_*s*1_ = 0.5, π_*o*2_ = 0.001, π_*o*1_ = 0.25). *First row:* dispositional priors on the first trial, shown as smoothed distributions for illustrative purposes. An *Integrated* prior is subsequently updated through learning (shown in supplemental Figure S2), while a *Bad* dispositional prior is rigid. *Second row:* Behavior prediction. *Third row:* Internal state inference. *Fourth row:* External state inference. *Fifth row:* Person state inference. *Column (a):* Inference with an integrated prior over internal states. *Column (b):* Inference with a latent prior that others are “all-bad”: after observing poor behavior the subject infers the other is a *Bad* person (fourth row, from trial 15 onward), after which predictions remain pessimistic; improved behavior is then attributed to a favorable external factor (or “ulterior motive”). *Column (c):* A *Bad* latent prior with higher prior probability: The subject switches more readily to infer the other is “all-bad.” See the online article for the color version of this figure.

As shown in Figure 5a, when the prior probability of a *Bad* person is zero (ψ_*Split*_ = 0), the subject tracks changes in behavior and attributes these evenly to changes in internal and external states. Figure 5b shows the predictions of a model that includes a low prior probability of a *Bad* person state (ψ_*Split*_ = 0.075). Here, the subject initially tracks changes in behavior, by updating an *Integrated* representation, as shown previously. However, as behavior worsens, the subject infers that they are dealing with a *Bad* person. Importantly, even when behavior subsequently improves, predictions remain pessimistic. Figure 5c shows model predictions with higher prior probability of a *Bad* person (ψ_*Split*_ = 0.25), where the subject more rapidly switches to inferring bad intent.[Fig fig5]

Once the subject has inferred that the other is a bad person, two processes tend to maintain this devaluation. First, local improvements in internal state are insufficient to overturn accumulated evidence for a global *Bad* disposition at the level above (see Figure 5b). Second, improved behavior is attributed to favorable external conditions (see Figure 5c). (We note here that favorable external conditions could equally be conceptualized as an additional internal factor in the form of an *ulterior motive*). Notably, these effects combine to consolidate devaluation over time, even in the face of countervailing evidence (Figure 5b, Row 5), by accumulating support for a *Bad* disposition through learning (The evolution of priors from trial-to-trial is shown in supplemental Figure S2). These findings accord with clinical and everyday observations that trust can be difficult to rebuild once ruptured (Hula et al., 2018; King-Casas et al., 2008) and suggest a mechanism by which polarized beliefs can increase in fixity (see Cook & Lewandowsky, 2016).

### Idealization and Devaluation With Split Dispositional Priors

**Figure 6 fig6:**
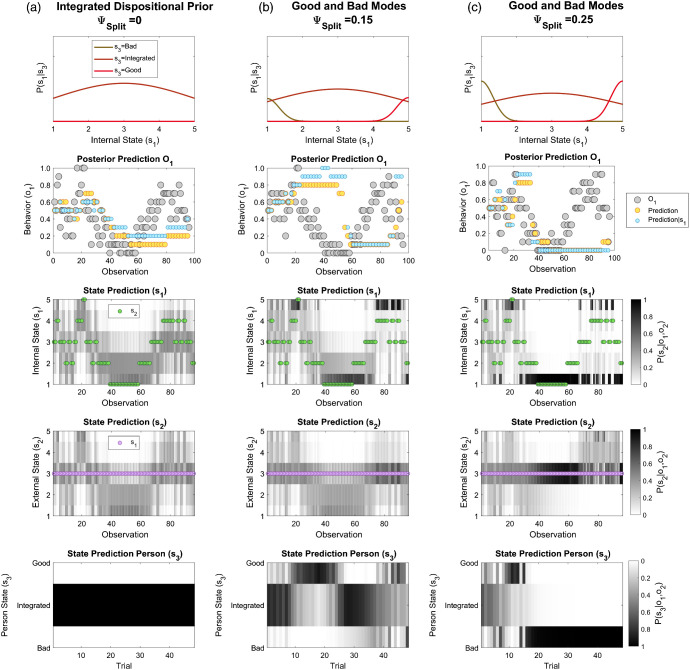
Inference With Latent “Bad” and “Good” Dispositional Priors *Note*. Simulated data are plotted as in Figure 5 (π_*s*1_ = 0.5, π_*o*2_ = 0.001, π_*o*1_ = 0.25). *Column (a):* Inference with an integrated (unimodal) prior over internal states. *Column (b)*: Inference with latent priors that others are either “all-bad” or “all-good” (ϕ_*Bad*_ = 0.5), resulting in phases of devaluation and idealization following bad or good observations, respectively. *Column (c):* When split latent priors have higher prior probability, bistable dynamics emerge. Internal state inference is initially flexible and veridical, but oscillates between extremes once split priors are activated, in this case culminating in stable devaluation after Trial 20. See the online article for the color version of this figure.

As shown in supplemental Figure S3, activation of a latent *Good* person state leads to stable idealization in an equivalent manner to that described above for devaluation. To illustrate switches from idealization to devaluation, we implement both negative and positive latent dispositions (ψ_*Bad*_ = 0.5). As previously, context cues are uninformative (π_*o*2_ = 0.001) and remaining parameters are configured as for previous simulations (π_*s*1_ = 0.5, π_*o*1_ = 0.25, ψ_*Ext*_ = 0.6). Integrated inference is shown for comparison (Figure 6a, ψ_*Split*_ = 0). Importantly, with increasing splitting, a subject is prone to draw more extreme inferences about others’ intentions (Figure 6b, ψ_*Split*_ = 0.1). As the prominence of splitting further increases, a subject may still be capable of learning about others, but becomes stuck in phases of idealization and devaluation after observing good or bad behavior, respectively (Figure 6c, ψ_*Split*_ = 0.25). As shown previously, during each phase, countervailing evidence is partly attributed to external factors.[Fig fig6]

Notably, since the model includes a small probability that intentions can change within a given epoch (trial), inferences about the other’s internal states were still prone to local oscillations, even where inference over a global disposition remained stable. For example, in Figure 6c (Row 5), after trial 20 the subject infers the other is a *Bad* person, though their inferred internal state (*s*_1_) nevertheless changes frequently (Figure 6c, Row 3). This arises since local evidence for good intentions (within a trial) is insufficient to overturn accumulated evidence (across trials) that the other is generally bad. Such a pattern resembles attributions seen clinically during devaluation, wherein improvements are discounted as “one-off” exceptions.

**Figure 7 fig7:**
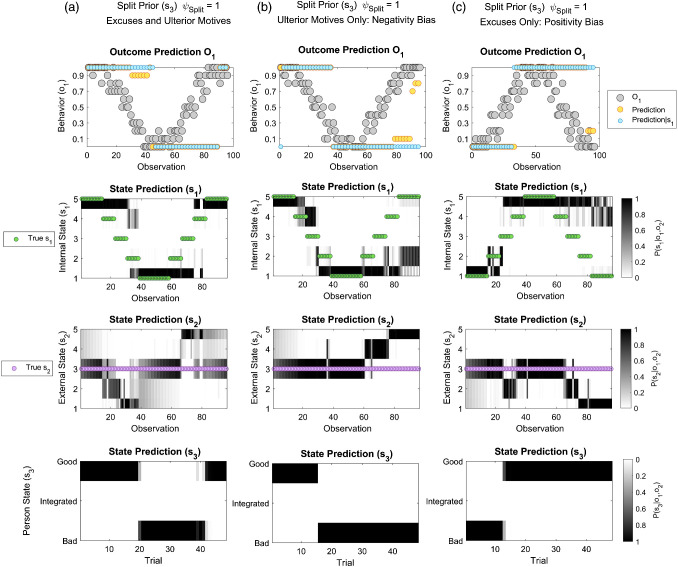
Splitting, Negativity, and Positivity Biases *Note*. Simulated data with split priors only (ϕ_*Bad*_ = 0.5, π_*o*2_ = 0.001, π_*o*1_ = 0.75). *Column (a):* Split inference with both Favorable and Unfavorable external states. The subject initially infers the other is Good, and attributes worsening behavior to Unfavorable external factors (i.e., an excuse). The subject switches to inferring Bad intent only after seeing “inexcusable” behavior. After switching to infer Bad intent, the subject attributes improving behavior to Favorable external factors (i.e., an ulterior motive), and switches back to inferring Good intentions only after seeing exemplary behavior. *Column (b):* With a Favorable external factor (“ulterior motive”) only, the subject switches readily to stable devaluation, corresponding to a negativity bias. *Column (c):* With an Unfavorable external factor only (“excuse”), the subject switches readily to stable idealization, corresponding to a positivity bias. See the online article for the color version of this figure.

A bistable pattern is illustrated by considering the extreme case of symmetrically split priors with no integration (ψ_*Split*_ = 1, ψ_*Bad*_ = 0.5, π_*o*2_ = 0.001, π_*o*1_ = 0.75). Here, after initially observing good behavior, the subject persists in inferring a person is *Good*, even after seeing several instances of mediocre behavior. Worsening behavior is explained away as due to unfavorable external conditions (i.e., “excuses”). Only after observing “inexcusably bad” behavior does the subject switch to infer a *Bad* person. The subject then persists in inferring that the other is *Bad*, even after seeing several instances of good behavior that would previously have supported a conclusion that they were *Good* (Figure 7a). Beyond this point, improvements in behavior are explained away as due to favorable external conditions (or an “ulterior motive”). The subject only switches back to inferring the other is *Good* after seeing extremely good behavior. This finding resembles an intuition that concrete gestures of “going the extra mile” can be required to recover relations following ruptures in cooperation in BPD (A. W. Bateman et al., 2015).[Fig fig7]

### Negativity and Positivity Biases

The relative stability of devaluation and idealization depends on the extent to which good and bad behavior, respectively, can be attributed to external (situational) factors. To illustrate this, we simulate split priors (ψ_*Split*_ = 1, ψ_*Bad*_ = 0.5, π_*o*2_ = 0.001, π_*o*1_ = 0.75), while changing the configuration of the external factor (see supplemental material). We first increase an effect of favorable external states on behavior, while removing an effect of unfavorable external states. Here, people with bad intentions may supply even very positive outcomes (e.g., to deceive) but people with good intentions do not supply negative ones (“no excuses”). This increases the range of behavior consistent with bad intent, thereby stabilizing devaluation. As shown in Figure 7b, the subject more readily changes impressions of another person from *Good* to *Bad* than *vice versa*, consistent with existing findings of a “negativity bias” in social inference (Amabile & Glazebrook, 1982; Brown et al., 2005; Hamilton & Zanna, 1972; for reviews, see Skowronski & Carlston, 1989; Ybarra, 2002; see General Discussion). This pattern also evokes a description of paranoid personality disorder, as characterized by “misconstruing the neutral or friendly actions of others as hostile or contemptuous,” and a “preoccupation with unsubstantiated ‘conspiratorial’ explanations of events” (World Health Organization, 1992). Conversely, increasing an effect of unfavorable external states, while removing an effect of favorable external states, increases the range of “excusable” behavior and enhances a tendency toward idealization (Figure 7c). Here, the subject exhibits behavior that is insensitive to negative consequences, consistent with a “positivity bias” (e.g., Wojciszke et al., 1993).

### Modifying Splitting: Psychotherapeutic Analoges

The stable phases of idealization and devaluation described above are underpinned by two key model features. First, information about the external context is sufficiently imprecise as to place few constraints on inference, thereby allowing explaining away. Second, split priors are impervious to learning, in so far as their extremity and precision is not modifiable. We conclude our illustration of the model by examining these two features, both of which are pertinent to psychotherapeutic interventions aimed at reducing splitting.

#### External Context Perception

**Figure 8 fig8:**
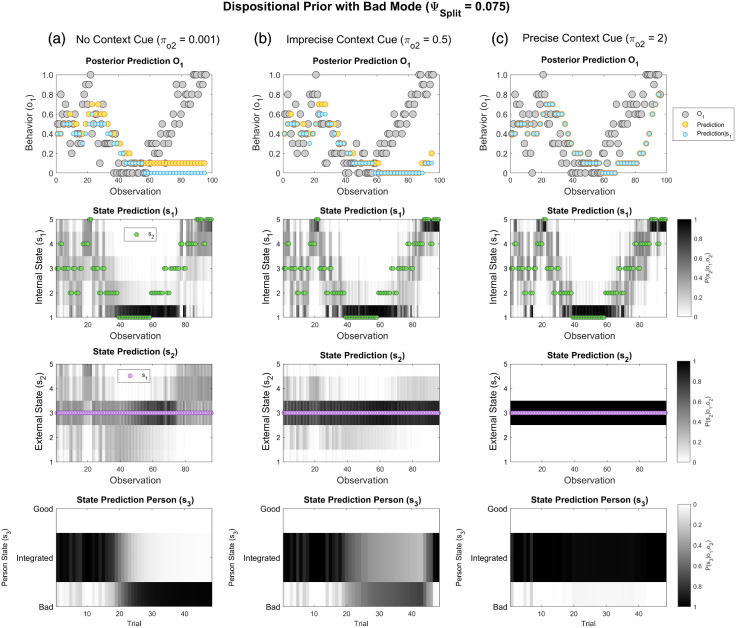
Context Information Ameliorates Splitting *Note*. Simulated data are plotted as in Figure 5b (ϕ_*Bad*_ = 1, π_*s*1_ = 0.5, π_*o*1_ = 0.25), for varying cue precision, π_*o*2_. Here, the other’s internal state (second row, green circles) changes over time. *Column (a):* Inference with a latent prior that others are “all-bad”: After observing poor behavior, the subject infers the other is a *Bad* person (fourth row, from trial 15 onward), after which predictions remain pessimistic. In the absence of informative external state cues, improved behavior is attributed to a favorable external factor (or “ulterior motive”). *Column (b):* With partial external state information devaluation still occurs, though recovery is possible (Trial 45 onward). *Column (c):* Reliable external state information prevents devaluation, despite veridical inference regarding the other’s transient bad intentions (observations 40–60). See the online article for the color version of this figure.

Idealization and devaluation can be ameliorated by increasing the precision with which the subject can perceive the external context, π_*o*2_, thereby promoting more veridical social inference. To illustrate this, we simulate devaluation with a *Bad* latent prior (ψ_*Bad*_ = 1, ψ_*Split*_ = 0.075, π_*s*1_ = 0.5, π_*o*1_ = 0.25), in a situation where the other’s internal state changes across time. The results are shown in Figure 8. Simulation with uninformative external state cues is shown for comparison (π_*o*2_ = 0.001; Figure 8a). With partial context information (π_*o*2_ = 0.5; Figure 8b) devaluation becomes reversible, given sufficient counter-evidence. Providing *precise* contextual information (π_*o*2_ = 2; Figure 8c) almost entirely prevents devaluation at this setting of ψ_*Split*_. This happens since speculative inference that the other’s actions are influenced by hidden external factors (e.g., an ulterior motive) is reduced, and the subject can therefore more accurately track the other’s Intentions using an *Integrated* representation.[Fig fig8]

As shown in supplemental Figure S4, similar effects are seen when the other’s *external* state changes across time (e.g., varying transport delays), with a *Bad* latent prior (ψ_*Bad*_ = 1, ψ_*Split*_ = 0.05, π_*s*1_= 0.5, π_*o*1_ = 0.25). With no contextual information (supplemental Figure S4a, π_*o*2_ = 0.001), the subject falsely attributes the other’s worsening behavior to their *Bad* disposition, rather than their adverse circumstances (e.g., “they are late because they hate me”). Introducing context information largely prevents this false inference (supplemental Figure S4b, π_*o*2_ = 0.25). By analogy, psychotherapeutic interventions might ameliorate splitting by promoting contextualized appraisals of others. Notably, however, if a propensity to devaluation is increased, context information is interpreted in a biased fashion (supplemental Figure S4c, ψ_*Split*_ = 0.25). Specifically, after a switch to devaluation has occurred, *unfavorable* context information is mistrusted (supplemental Figure S4c, observations 30–70; e.g., “I don’t believe the train was delayed—the person is late because they hate me”). The latter effect arises due to an expectation, built into the model, that the behavior of *Bad* people is inexcusable (see “Second-level likelihoods” in supplemental material). Thus, higher degrees of splitting may distort inference about external reality, potentially limiting the efficacy of psychotherapy in situations where splitting is more profound.

#### Modifying Split Priors

As described above, under the model, a subject can learn through experience the relative frequency with which splitting and integration best explain their observations. Such “high level” learning updates a person prior, *P*(*s*_3_), while leaving the form of split dispositional priors unmodified. Latent split priors can therefore be reinstated, for example, in response to unexpectedly extreme observations (cf. Dunsmoor et al., 2015; Gershman & Niv, 2012). From a psychotherapeutic perspective, a person with such a model remains vulnerable to splitting when conditions markedly worsen or improve.

An alternative arrangement would allow split priors themselves to be updated. To explore this latter possibility, we relax an assumption that the Dirichlet distributions governing $P\left(s_{1}|s_{3}=Bad\right)$ and $P\left(s_{1}|s_{3}=Good\right)$ are based on a large number of previous observations. Instead, we set the number of previous observations to a small number (10), meaning that split priors can, in principle, readily change in response to new observations. As shown in supplemental Figure S5, this arrangement also gives rise to idealization and devaluation. However, idealization and devaluation become less marked across time. This results since *Bad* and *Good* priors, though initially stabilized by external attributions, are eventually modified through learning (illustrated in supplemental Figure S6). Such “low-level” learning can prevent reinstatement of splitting, and therefore appears desirable from a psychotherapeutic perspective.

## Predictions of a Split-HMM

A key feature of the split-HMM is that extreme outcomes are prone to trigger latent splitting, and therefore tend to “freeze” learning. An ensuing prediction is that *negative* observations entrain more confident, rigid beliefs to the extent that a *Bad* person prior is present, while *positive* observations entrain more confident, rigid beliefs to the extent a *Good* person prior is present. Thus, distinct from previous descriptive models, a split-HMM makes quantitative predictions regarding the rate of belief updating following negative and positive observations.

### Simulating Differential Belief Updating From “Bad” and “Good” Observations

To illustrate effects of splitting on learning rate, we simulated responses to objectively “bad” and “good” agents as the relative prominence of *Bad* and *Good* person priors changes. For a “bad” agent, we arranged simulations such that internal state started at *s*_1_ = *Moderately Bad*; for a “good” agent, internal state started at *s*_1_ = *Moderately Good*. For both agents, internal state subsequently evolved on a discrete random walk with a low level of volatility and zero net drift; across simulations therefore, the “bad” agent exhibits uncharitable behavior on average, while the “good” agent exhibits charitable behavior on average. We set ψ_*Split*_ = 0.05 and gradually increased ψ_*Bad*_ from 0 to 1. At each parameter setting, we simulated responses to 240 sets of 96 observations sampled from each agent.

Under a split-HMM, a nonparametric probability distribution over hidden states is updated after each observation. By contrast, relevant existing studies of social inference (Diaconescu et al., 2020; Siegel et al., 2018, 2020) use models based on point estimates (e.g., Rescorla-Wagner learning) or parametric estimates (e.g., a mean and variance; Mathys et al., 2011). These studies express changeability of predictions as a learning rate, conventionally denoted as α, where a higher learning rate entails faster updating in response to unexpected observations. To quantify belief updating in a split-HMM in equivalent terms, we calculate a learning rate using maximum *a posteriori* predicted outcomes under the model as point estimates. To do so, we leverage a canonical definition of learning rate, α, as the slope of a relationship between changes in predictions and prediction errors across observations (see supplemental material). (Note that α is distinct from the learning parameters used to update Dirichlet distributions, which are fixed). We also express uncertainty in beliefs as the Shannon entropy of a posterior distribution over internal states, *s*_1_.

**Figure 9 fig9:**
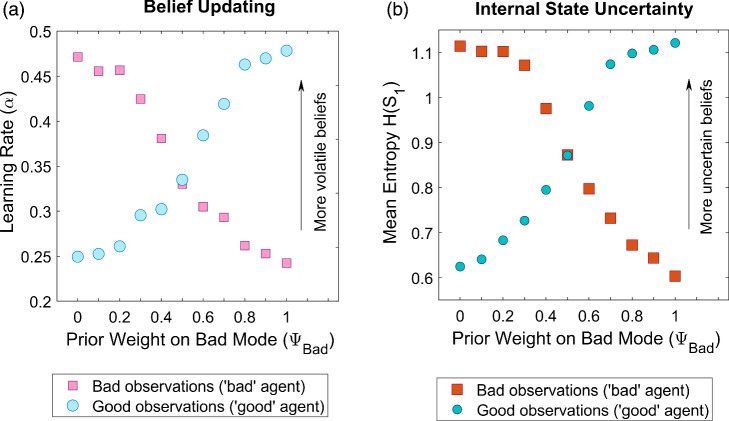
Model Predictions: Differential Learning From Good and Bad Observations *Note*. Net learning rate and internal state uncertainty of a model with latent splitting for observations generated from “bad” and “good” agents (see Main Text; π_*o*2_ = 0.001, π_*o*1_ = 0.25, π_*s*1_ = 0.5, ψ_*Split*_ = 0.05, ψ_*Ext*_ = 0.6). (a) Learning rates for a “bad” agent decrease, and learning rates for a “good” agent increase as the prior probability of a latent Bad mode, ψ_*Bad*_, increases. (b) Uncertainty over internal state decreases for a “bad” agent and increases for a “good” agent as the prior probability of a latent Bad mode, ψ_*Bad*_, increases. In summary, a more prominent Bad latent mode, (ψ_*Bad*_ > 0.5) entrains rigid, pessimistic beliefs in response to bad observations, while a more prominent Good latent mode, (ψ_*Bad*_ < 0.5) entrains rigid, optimistic beliefs in response to good observations. See the online article for the color version of this figure.

As expected, learning rate, α, for the “bad” agent decreased monotonically as a *Bad* latent prior increased in prominence, that is, as ψ_*Bad*_ increased (Figure 9a). A symmetric effect was seen for responses to the “good” agent as a *Good* latent prior increased in prominence, that is, as ψ_*Bad*_ decreased (Figure 9a). The same pattern was seen for uncertainty over internal states: uncertainty for the “bad” agent decreased monotonically as a *Bad* latent prior increased in prominence, and similarly decreased for the “good” agent as a *Good* latent prior increased (Figure 9b). (As shown in supplemental Figure S7, increasing ψ_*Split*_ increases the convexity of these relationships).[Fig fig9]

### Evidence for Differential Belief Updating

Recent studies of moral inference report differential belief updating and uncertainty for “bad” and “good” agents consistent with the predictions described above (Siegel et al., 2018, 2020). In such studies, participants rated the moral character of two agents, after observing the extent to which each agent was willing to accept money to deliver painful electric shocks to a third person (Siegel et al., 2018): a “bad” agent was more inclined to take money at the expense of shocks for the other person, while a “good” agent was more charitable. Participants were found to more rapidly update their predictions about bad rather than good agents and were also *more uncertain* in their appraisals of the moral character of bad agents than of good agents.

To explain the above findings, Siegel et al. (2018) suggested that observing bad behavior primes feelings of threat, causing beliefs about others to become more uncertain and therefore more amenable to rapid updating. In support of this idea, observing bad behavior increased the rate of belief updating for subsequent, unrelated judgments of competence (Siegel et al., 2018, Study 5). Siegel et al. (2018) showed that a combination of faster updating and greater uncertainty is consistent with a Bayesian learning model wherein “bad” agents are perceived as more volatile. Such flexibility is said to promote vigilance against worsening behavior, while also allowing beliefs to be amended if behavior improves, thereby supporting recovery of cooperation. Using the same experimental design, Siegel et al. (2020) found that participants with BPD exhibited less asymmetry in beliefs about “bad” and “good” agents compared with non-BPD control participants. The authors proposed that people with BPD lack the adaptive mechanism to increase volatility following bad observations, resulting in more rigid beliefs about “bad” others, and thereby slowing recovery from ruptures of trust.

A split-HMM offers an alternative explanation, based on differing latent priors in BPD and non-BPD participants, with emergent effects on learning rate and uncertainty. In particular, if non-BPD participants were to hold a latent prior that others tend to be *Good* (ψ_*Bad*_ < 0.5), this would lead to a higher learning rate and greater uncertainty for “bad” than for “good” others (as shown in Figure 9). Furthermore, if people with BPD were to have more symmetric split (latent) prior expectations about others’ moral character, that is, ψ_*Bad*_ ≈ 0.5, than non-BPD participants, this would account for their more symmetric belief updating for “bad” and “good” agents, especially at higher settings of ψ_*Split*_. Strong ensuing predictions are that ψ_*Bad*_ and ψ_*Split*_ estimated from ratings of moral character are greater for BPD participants than for non-BPD participants, and that for non-BPD participants, ψ_*Bad*_ is significantly less than 0.5. A further prediction is that distributions of prior moral character ratings made by BPD and non-BPD participants are significantly different.

## Estimating Splitting From Moral Inference Data

To test the above predictions, and to illustrate how the split-HMM can be used to derive empirical estimates of splitting, we fit the model to data from the study of Siegel et al. (2020), kindly made available by the authors. Using existing data to test the model not only makes efficient use of scientific resources, but also mitigates potential experimenter-induced bias, since the former study was not carried out with the current model in mind. In our view, these advantages outweigh limitations arising from the fact that the study is not optimized to test all aspects of the split-HMM (see General Discussion).

In brief, the aforementioned study required participants to learn about the moral character of “bad” and “good” simulated agents by observing how agents behaved toward a third party. Participants made serial appraisals of the moral character of the two agents and reported their uncertainty about these appraisals, corresponding to internal state inference in the split-HMM. Furthermore, participants made moral character ratings before observing the behavior of either agent, corresponding to dispositional priors in the split-HMM. The data set is therefore highly suitable for testing the quantitative predictions outlined above.

### Experimental Paradigm

**Figure 10 fig10:**
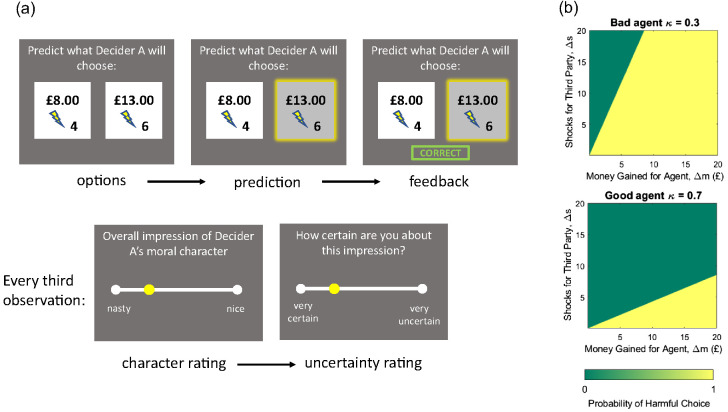
Design of Moral Inference Task (Siegel et al., 2020) *Note*. Reproduced from “A Computational Phenotype of Disrupted Moral Inference in Borderline Personality Disorder,” by J. Z. Siegel, O. Curwell-Parry, S. Pearce, K. E. A. Saunders, and M. J. Crockett, 2020, *Biological Psychiatry: Cognitive Neuroscience and Neuroimaging*, *5*(12), pp. 1134–1141 (https://doi.org/10.1016/j.bpsc.2020.07.013). (a) Moral inference task: participants predicted choices made by two agents (Decider A and Decider B) between two options: more shocks inflicted on a third party in exchange for more money for the agent or fewer shocks for the third party in exchange for less money for the agent. After each prediction, the agent’s actual choice was revealed, followed by feedback indicating whether the participant’s prediction was correct or incorrect. After every three observations, participants rated the agent’s moral character (ranging from nasty to nice), and how certain they were about their impression. (b) Exchange rates between money and shocks for the two agents: a “bad” agent was more willing to inflict shocks to obtain money, while a “good” agent was more charitable. Copyright 2020 by the Society of Biological Psychiatry. Reproduced by permission. See the online article for the color version of this figure.

The task design is illustrated in Figure 10a. Participants observed choices made by two agents (called “Decider A” and “Decider B”). On each of 48 choices, the observed agent selected one of two options, each of which entailed an amount of money for themselves and a number of painful electric shocks for a third person. Participants observed choices made by the two agents in series; agent order was counterbalanced across participants. Before observing an agent’s choices, participants rated both the agent’s expected moral character, and their uncertainty about this judgment (on scales from 0 = *nasty* to 100 = *nice* and 0 = *very uncertain* to 1 = *very certain*, respectively). These initial ratings indicated participants’ prior beliefs about people’s moral character. Participants were then asked to predict how the agent would choose at each timestep, after which the agent’s true choice was revealed. Subsequent moral character and uncertainty ratings were elicited after every three choices.[Fig fig10]

Agents were programmed to choose according to a “harm aversion” parameter, κ, where κ = 1 denotes minimizing shocks for the other person, and κ = 0 denotes maximizing money for oneself. Specifically, the subjective value to an observed agent of choosing the more harmful of the two options is given by:

$$V_{harm}(\kappa )=(1-\kappa )\Delta_{m}-{\text{κΔ}}_{s},$$ 4

where Δ_*m*_ and Δ_*s*_, respectively, represent the difference in money (for the agent) and shocks (for a third party) between the two choice options. A “bad” agent was inclined to maximize money (κ = 0.3), while a “good” agent was inclined to minimize shocks (κ = 0.7; shown in Figure 10b). Choice options were arranged such that each agent made a number of both harmful and helpful choices (see Siegel et al., 2020, for details).

Three groups of participants were tested by Siegel et al. (2020): a group with diagnoses of BPD (*N* = 20), a group with diagnoses of BPD who had completed a democratic therapeutic community treatment (“DTC,” *N* = 23), and a control group without BPD diagnoses (“non-BPD,” *N* = 102), matched to the BPD group on age (±4 years), gender, and education. BPD participants were recruited from an outpatient population, and diagnosis was confirmed through a structured clinical interview. Non-BPD participants were shown the same sequence of observations as their matched BPD counterparts. We refer the reader to Siegel et al. (2020) for further details of participant recruitment and experimental design.

### Model Fitting Methods

Siegel et al. (2020) modeled participants’ choice predictions. Here, by contrast, we focus on fitting moral character ratings, since these correspond closely to the internal state dimension of our model. We assume that subjects infer an agent’s degree of harm aversion by inverting a generative model of the agent’s choices. We assume also that subjects report moral character ratings by sampling internal states from the same generative model.

#### Internal States

We implement a generative model with a one-to-one mapping between internal hidden states, *s*_1_, and expected harm aversion, μ = E[κ], ranging from 0 to 1 in increments of 0.1. The model incorporates noise in a subject’s model of an agent’s choices. Specifically, if the model is run forward, each *s*_1_ emits a setting of κ, drawn from a Gaussian likelihood distribution with mean μ and precision π_*o*1_. For instance, *s*_1_ = 2 entails κ ∼ *N*(μ = 0.1, σ^2^ = 1/π_*o*1_) (Gaussian likelihoods are truncated such that 0 < κ < 1). Given an emitted κ, an agent selects the money-shocks option with the highest subjective value (see supplemental material; for a similar random preference model, see Moutoussis et al., 2016). The degree of emission noise is governed by the first-level precision, π_*o*1_. In simple terms, an agent’s choices provide noisy estimates of their underlying propensity to harm. Subjects are equipped with integrated and split second-level priors as described previously. We arrange the model timescale to match that of the experiment, with three first-level epochs per trial, after which priors are updated to mediate learning.

#### External States

Although the experiment provides no explicit external context for agents’ choices, we nevertheless incorporate an external state dimension when fitting the model. In other words, we allow the subject to attribute changes in the agents’ observed behavior to unobserved external factors. Here, favorable external states bias upward the expected values of harm aversion, μ, for each level of internal state. Similarly, unfavorable external states bias downward expected values of harm aversion. An external state dimension thus allows for the possibility that an agent’s manifest behavior is not a veridical reflection of their character, but rather subject to hidden external constraints. For instance, a participant might suppose that an ostensibly “bad” agent has been instructed to behave badly by the experimenter. Since no external state information was available to the participants, we set π_*o*2_ = 0.001 when fitting the model.

#### Model Fitting Routine and Parameters

To fit the model, we assume that participants report moral character ratings, *ŝ*_1_, by sampling from their generative model. To obtain a likelihood function, we first discretize moral character ratings across eleven bins, matching the scale of *s*_1_. We then treat these discretized ratings as samples from a participant’s posterior belief about the agent’s harm aversion. We find model parameters for each participant that maximize this likelihood function, using a bounded optimization routine in MATLAB (*fmincon*, Mathworks, Provo, UT). A single set of parameters were used to fit a participant’s responses to both “bad” and “good” agents. We consider nested models with up to five free parameters, as defined previously: π_*o*1_, π_*s*1_, ψ_*Bad*_, ψ_*Split*_, and ψ_*Ext*_. We test restrictions in which ψ_*Split*_ = 0 (integrated priors only) and in which ψ_*Split*_ = 1 (split priors only). We compare nested split-HMM using likelihood ratio tests, using the mean log-likelihood ratio (log LR) across participants between restricted and unrestricted models. Following the approach taken by Siegel et al. (2020), we compare mean model parameters between BPD and non-BPD groups, to test for an effect of diagnosis, and between BPD and DTC groups, to test for an effect of treatment.

#### Hierarchical Gaussian Filter Model

Siegel et al. (2020) fitted participants’ choice predictions using a Bayesian model with an adaptive learning rate, the Hierarchical Gaussian Filter (HGF; Mathys et al., 2011). Under this model, the learning rate depends on volatility in a latent state governing an expectation over κ (equivalent to *s*_1_ here). Here, we fit an HGF model to *moral character ratings*, following the general procedure described by Siegel et al. (2020; see supplemental material). This model has four free parameters: a log volatility and a choice stochasticity parameter for each agent. We perform model comparison between the HGF model and the split-HMM as competing accounts of behavior, using the Bayesian information criterion (BIC). We configure the HGF with the same set of priors used by Siegel et al. (2020). The parameters of each model, and criteria for model comparison, are summarized in supplemental Table 1.

### Data and Code Availability

Computer code used for model fitting is available on request to the authors. Previously published data analyzed in this study are available on request to the original authors, Siegel et al. (2020).

### Results

#### Prior Subjective Moral Character Ratings

**Figure 11 fig11:**
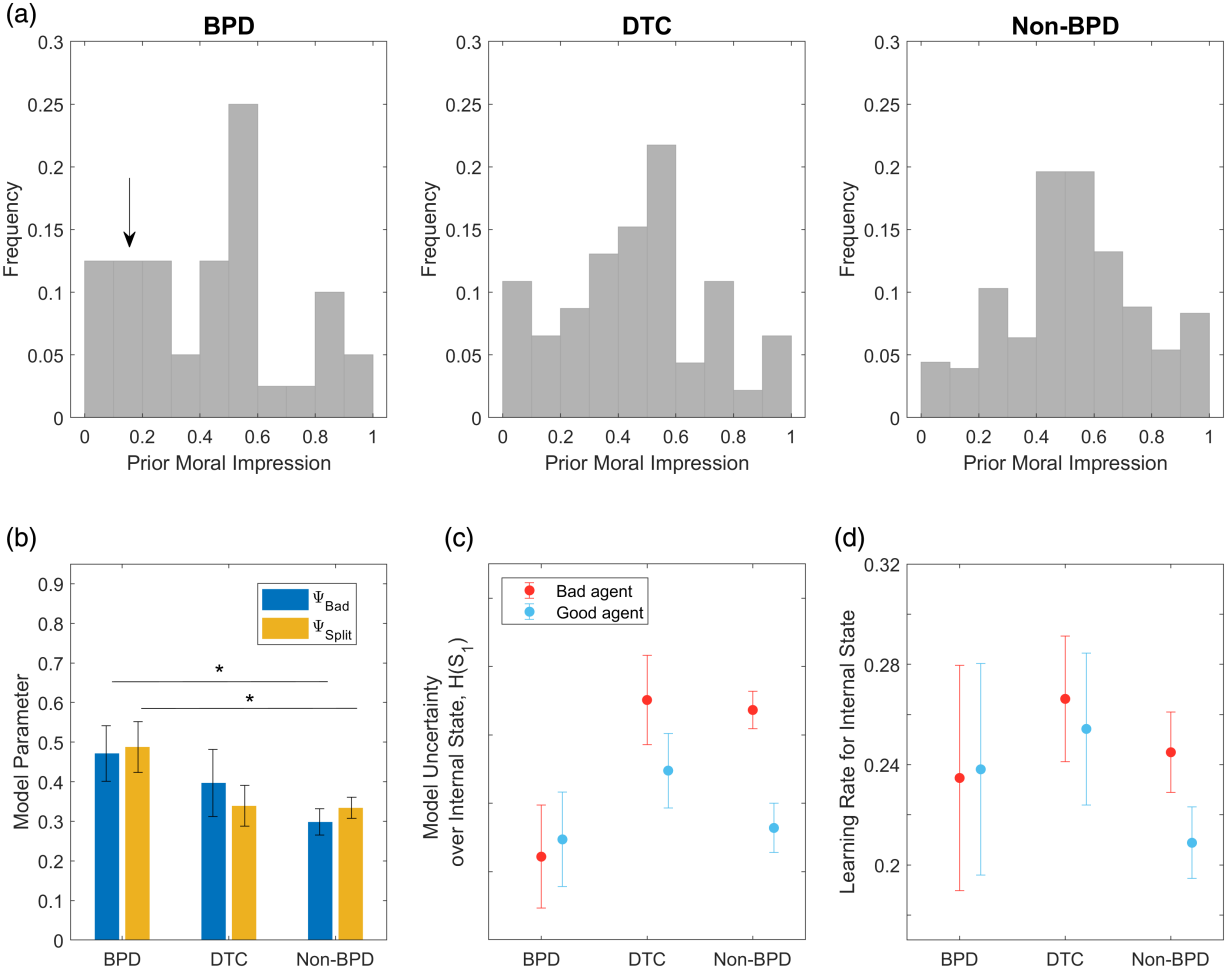
Prior Moral Character Ratings, Model Parameters, and Predictions *Note*. (a) Observed histograms of prior moral character ratings (combined across agents) within each participant group. Prior ratings at the negative end of the distribution are particularly prominent in the BPD group (*N* = 20) by comparison with non-BPD participants (*N* = 102; marked with an arrow). The distribution in the DTC group (*N* = 23) appears intermediate between BPD and non-BPD groups. (b) Comparison of best fit parameters for a split-HMM across groups: ψ_*Bad*_ and ψ_*Split*_ are significantly greater in BPD than in non-BPD participants. (c) Model-derived internal state uncertainty across participant groups. (d) Model-derived learning rate across participant groups. The split-HMM reproduces an effect previously reported, wherein non-BPD participants exhibit greater learning rate and uncertainty for “bad” as opposed to “good” agents, an effect that is attenuated in BPD participants. HMM = hidden Markov model; BPD = borderline personality disorder; DTC = democratic therapeutic community. See the online article for the color version of this figure. *Significance at *p* < .05.

Each participant made prior moral character ratings before observing each agent’s choices. Distributions of the prior moral character ratings made by participants in each group, concatenated across agents, are shown in Figure 11a. Prior ratings in the non-BPD group appear predominantly unimodal. By contrast participants with BPD show an apparently trimodal distribution, with more extreme prior ratings at both poles. As marked in Figure 11a, prior ratings at the negative end of the distribution are particularly prominent in the BPD group by comparison with non-BPD participants. Each participant provides only a prior point-estimate, rather than a distribution. Nevertheless, the observed group-level distributions approximate the form of latent priors postulated by the model.[Fig fig11]

A Kolmogorov–Smirnov (KS) test rejects the null hypothesis that prior ratings made by BPD and non-BPD, concatenated across agents, were drawn from the same underlying distribution (*p* = .038, KS = 0.24). The same result is found if the analysis is restricted to the first rating made by each participant, before encountering either agent (*p* = .045, KS = 0.33). Participants in the DTC group show a distribution that appears intermediate between non-BPD and BPD groups, however, is not significantly different from the BPD group (first ratings, *p* = .724, KS = 0.20).

#### Model Comparison

Model comparison results are summarized in supplemental Table 1. A split-HMM with *Bad*, *Good*, and *Integrated* person states outperformed a restricted model with only an *Integrated* person state, mean ΔBIC = 18.0; mean log LR = 12.5; likelihood ratio test: χ^2^(2) = 25.0 *p* < .0001, and also outperformed a restricted model with only *Bad* and *Good* person states, mean ΔBIC = 135; mean log LR = 71.1; χ^2^(2) = 142.1, *p* < .0001. The best-fitting split-HMM also performed significantly better than a null model in which ratings were selected randomly, mean log LR = 17.9; likelihood ratio test: χ^2^(5) = 35.8 *p* < .0001. The same results obtained whether model comparison was performed for all participants as above, restricted to non-BPD participants, or restricted to BPD participants. This suggests that participants across groups were capable of learning the agents’ moral character, but were also prone to excessively extreme character ratings to varying extents. For the best-fitting model, Mcfadden’s pseudo-*R*^2^ = 0.22, indicating a good fit to the data.

As reported previously, an HGF model with agent-specific volatility parameters can also account for higher learning rates and greater uncertainty for “bad” agents (Siegel et al., 2018, 2020). However, as shown in supplemental Figure S8, such a model fitted to moral character ratings accounts neither for the observed distribution of ratings, nor the optimistic character ratings made by non-BPD participants. In keeping with this, a split-HMM *Bad*, *Good*, and *Integrated* person states outperforms an HGF model in accounting for moral character ratings (total ΔBIC = 1827, mean ΔBIC = 12.6).

#### BPD Participants Exhibit Higher Splitting

Within the best-fitting split-HMM, we compared ψ_*Bad*_ and ψ_*Split*_ parameters between BPD and non-BPD groups, and between BPD and DTC groups (group means shown in Figure 11b). As predicted, both parameters were significantly greater in BPD participants than in non-BPD participants, two sample *t* test: ψ_*Bad*_: *t*(120) = 2.14, *p* = .034; ψ_*Split*_: *t*(120) = 2.34, *p* = .021. This finding supports an hypothesis that BPD participants are prone to more extreme evaluations of others’ character, and are more pessimistic in their appraisals of others than are non-BPD controls. The parameters of DTC participants were intermediate between BPD and non-BPD groups, but not significantly different from the BPD group at *p* < .05, ψ_*Bad*_: *t*(41) = 0.66, *p* = .509; ψ_*Split*_: *t*(41) = 1.83, *p* = .075. Parameter recovery for these two parameters was good (Pearson *r* between simulated and fitted parameters = 0.64 and 0.74 for ψ_*Bad*_ and ψ_*Split*_, respectively; supplemental Figure S9). Distributions of parameters across participants, and scatterplots showing relationships between ψ_*Split*_ and ψ_*Bad*_ in each group, are shown in supplemental Figure S10.

#### Splitting Accounts for Effects of Agent on Learning Rate and Uncertainty

We find that a *Good* person state for non-BPD participants accounts for their higher learning rate for “bad” relative to “good” agents. As shown in Figure 11c, in non-BPD participants, an estimated learning rate (α), based on maximum *a posteriori* model predictions, is higher for “bad” than “good” agents, consistent with previous findings (Siegel et al., 2020). Comparing learning rate across non-BPD and BPD groups, we find a main effect of agent, *t*(241) = 11.8, *p* < .0001, and a Significant Agent × Group Interaction, *t*(241)=−3.59, *p* = .0004, driven by a smaller effect of agent in BPD participants (Figure 11c). Here, more symmetric splitting for BPD participants accounts for a similar learning rate across both agents.

As shown in Figure 11d, the model also reproduces the differential effects of agent on uncertainty reported by Siegel et al. (2020). Positive splitting for non-BPD participants accounts for their higher uncertainty for “bad” relative to “good” agents; this arises since “good” agents tend to activate a *Good* dispositional prior. By contrast, BPD participants show similar internal state uncertainty for both agents, consistent with splitting.

#### A Split-HMM Accounts for Posterior Distributions of Character Ratings

**Figure 12 fig12:**
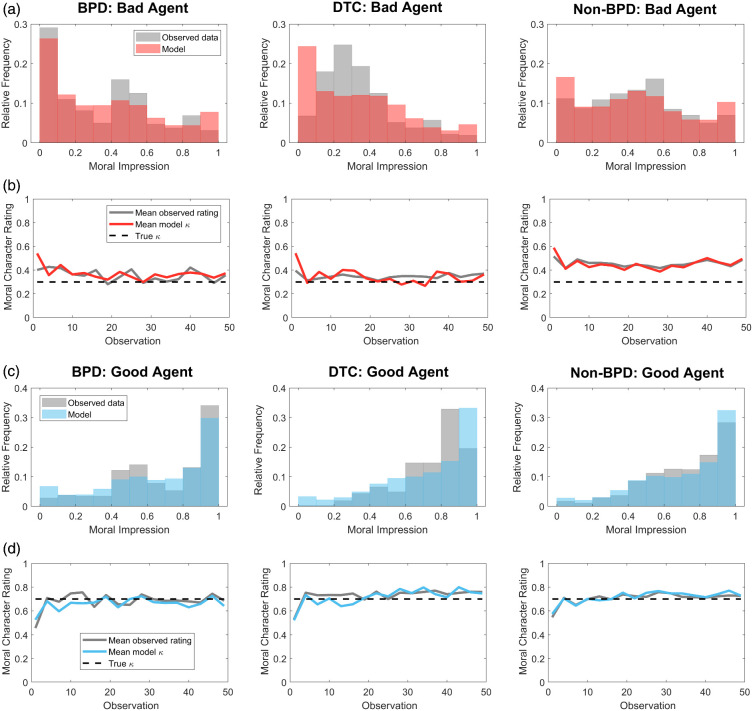
Posterior Moral Character Ratings and Model Fits *Note*. (a) Histograms showing distributions of posterior moral character ratings of the “bad” agent across all trials for each group of participants. Non-BPD participants (*N* = 102) show a predominantly unimodal distribution of ratings for the “bad” agent. By contrast, BPD participants (*N* = 20) make more extreme ratings. (b) Mean character ratings of the “bad” agent across observations. Ratings of the “bad” agent made by non-BPD participants are optimistic relative to the true κ. (c) Distributions of posterior moral character ratings of the “good” agent across all trials for each group of participants. Ratings made by BPD participants appear more concentrated at a positive extreme. (d) Mean character ratings of the “good” agent across observations. Across all groups, mean ratings for the “good agent” accurately converge on the true setting of κ. The above effects are reproduced by a split-HMM. HMM = hidden Markov model; BPD = borderline personality disorder; DTC = democratic therapeutic community. See the online article for the color version of this figure.

Notably, model parameters indicate that non-BPD participants showed a positive bias, with ψ_*Bad*_ significantly less than 0.5, mean ψ_*Bad*_ = 0.30, *t*(101) = −6.10, *p* < .0001. By contrast, BPD participants showed a more symmetric pattern of splitting, with ψ_*Bad*_ not significantly different from 0.5, mean ψ_*Bad*_ = 0.47, *t*(101) = −0.40, *p* = .69. As shown in Figure 12, these effects are visible in the distribution of posterior character ratings made by each group. The non-BPD group show a relatively symmetric, unimodal distribution of ratings for the “bad” agent (Figure 12a), and their ratings of the “bad” agent converge to an estimate that is optimistic relative to the true κ (Figure 12b). By comparison, BPD participants make more extreme ratings of the “bad” agent, which on average converge to an estimate close to the true κ.[Fig fig12]

For the “good” agent, ratings made by all three participant groups are positively skewed (Figure 12c), though ratings made by BPD participants appear more concentrated at the extreme. Across all groups, mean ratings for the “good” agent accurately converge on the true setting of κ (Figure 12d). As shown in Figure 12, these effects are reproduced by the model. Optimistic posterior ratings of non-BPD participants result from lower splitting, with a bias toward a *Good* dispositional prior.

## General Discussion

In this article, we model “splitting,” or dichotomous thinking, from a Bayesian perspective. In keeping with previous approaches (e.g., Ajzen & Fishbein, 1975; Diaconescu et al., 2020; Moutoussis, Fearon, et al., 2014; Reiter et al., 2019; Siegel et al., 2018), a subject learns about others’ dispositions by accruing information about their behavior across time. A novel feature is the addition of latent, split representations of others’ dispositions as either extremely good or extremely bad, whose likelihood is increased following “good” or “bad” observations, respectively. The resulting extreme beliefs resist counter-evidence through attribution to external-situational factors: During idealization, negative surprises are attributed to unfavorable external conditions (“excuses”), while during devaluation, positive surprises are attributed to favorable external conditions (with “ulterior motives”). However, if sufficient counter-evidence is observed, split beliefs can undergo precipitous changes of polarity.

A quantitative prediction of the model is that splitting tends to slow learning. Thus, to the extent that a *Bad* dispositional prior is prominent, subjects are prone to learn less quickly after exposure to negative environments, since they tend to become stuck in devaluation. Conversely, to the extent that a *Good* dispositional prior is prominent, subjects are prone to learn less quickly after exposure to positive environments, since they tend to become stuck in idealization. We have shown that these effects can account for an existing finding that healthy participants hold more certain, less malleable beliefs about “good” than “bad” others (Siegel et al., 2018, 2020), in terms of a latent prior that others are entirely good. In support of this hypothesis, the model reproduces the observed distribution of beliefs among healthy participants about the moral character of “good” and “bad” others.

### A Split-HMM Accounts for Moral Inference in BPD

Through simulation, we have shown how the split-HMM encompasses switches between idealization and devaluation that are consistent with a pattern of “unstable and intense interpersonal relationships” seen in BPD (American Psychiatric Association, 2013). We have also illustrated how a split-HMM can be fitted to experimental data to derive idiosyncratic estimates of splitting, and to distinguish participants with BPD from non-BPD participants cross-sectionally. By comparison with a non-BPD group, participants with BPD show less asymmetry in the fixity of their appraisals of “good” and “bad” others (Siegel et al., 2020). We show that this finding is explicable by latent beliefs among BPD participants that others can be either entirely good or entirely bad. In support of this hypothesis, character ratings made by BPD participants are concentrated at the extremes. Model fits accordingly reveal significantly greater splitting (and more symmetric splitting) in BPD participants than in non-BPD participants.

### Toward a Dimensional View of Relational and Affective Instability

At the time of writing, there has been a move toward a dimensional, rather than categorical, classification of personality pathology (Bach & First, 2018). Here, we have referred to specific personality disorders; however, the proposed model lends itself to a dimensional approach. In particular, we have shown how social inference can be parameterized along dimensions of splitting (splitting vs. integration), attribution (internal/dispositional vs. external/situational), and valence (negative vs. positive poles). We consider how the split-HMM might be used to assess each of these dimensions in turn below.

#### Splitting Versus Integration

Within the split-HMM, the relative prominence a priori of splitting versus integration is controlled by a free parameter, ϕ_*Split*_. A feature of the model is that the subsequent balance of integrated as opposed tosplit representations is adjusted through learning. Thus, split representations can stabilize and strengthen over time. We have shown that this is particularly likely to occur where others’ behavior is more extreme, and where the contextual causes of behavior are poorly signaled. The model is therefore congruent with an idea that splitting derives in part from an invalidating or emotionally impoverished childhood environment, as described by the developmental theories considered in the introduction (Bender & Skodol, 2007; Crowell et al., 2009; Fonagy & Luyten, 2009; Kernberg, 1967). We suggest that a baseline propensity to splitting, ϕ_*Split*_, might be an important dimensional marker of relational and emotional instability.

A further feature of the model is that, since split representations are themselves rigid (i.e., their form is not modified through learning), they can be *reinstated* if conditions become more extreme. This accords with an idea in psychoanalytic theory that splitting exists “in equilibrium” with more integrated functioning (Bion, 1963; Steiner, 1987, 1992). An alternative model would allow split priors themselves to be updated. We have shown that such learning tends to ameliorate splitting across time, and therefore appears desirable from a psychotherapeutic perspective.

#### Dispositional Versus Situational Attribution

We have shown that, when a subject is faced with unexpected observations, rather than change their polarized view of self or other, the subject finds it plausible to adopt a *more complex* explanation, one that depends on poorly observed external-situational causes (see Gershman, 2019). The excessive *precision* of split priors makes such external attributions more likely (see Kim et al., 2020). The split-HMM measures an individual’s general propensity toward external-situational attributions as a free parameter, ϕ_*Ext*_. We note that a prevailing tendency to privilege dispositional over situational attributions, classically referred to as a “fundamental attribution error,” might be taken to imply that low settings of ϕ_*Ext*_ are the norm (see, Harvey et al., 1981). However, the data examined here do not allow a trait-level tendency toward situational attributions (ϕ_*Ext*_) to be reliably estimated (supplemental Figure S9).

Future experimental work might incorporate an external state dimension into the design of a social inference task, to probe associated attributions. Relevant here are existing studies examining learning across more than one state dimension. For example, a recent study used a reinforcement learning paradigm, wherein outcomes could be subject to outside interference (Dorfman et al., 2019). The authors showed that participants indeed took into account external causes that could explain outcomes. In further example, Henco et al. (2020) tested a probabilistic learning paradigm in which participants received two cues indicating which of two playing cards was more likely to be rewarded: a “social” cue consisted of a face, whose eye gaze was directed to one of the two cards, while a “nonsocial” cue was provided by card color. Importantly, the helpfulness of social and nonsocial cues varied independently across trials, allowing the authors to measure the extent to which participants’ belief updates were influenced by social and nonsocial information. A similar design might be used to test the predictions of a split-HMM regarding how split inference over dispositional (“social”) factors influences attribution to situational (“nonsocial”) factors.

In the aforementioned study, Henco et al. (2020), using an HGF model, found that BPD participants placed higher weighting on social cues relative to healthy controls (an effect that was also seen among a participant group with diagnoses of schizophrenia). Relative to healthy controls, BPD participants were also more sensitive to volatility in cue-reward contingencies, for both social and nonsocial cues. That is, compared with controls, BPD participants showed greater adjustments in learning rate in response to changes in volatility. These findings are consistent with theories suggesting that people with BPD show heightened interpersonal sensitivity (Gunderson & Lyons-Ruth, 2008).

Further investigation is needed to examine how heightened interpersonal sensitivity in BPD might coexist with the bistable pattern of idealization and devaluation modeled here, which entails a degree of *insensitivity* to overt behavior during stable phases. It is noteworthy here that a propensity to attribute anomalous observations to extraneous factors, captured by ϕ_*Ext*_, influences the dynamic expression of splitting. A bias toward dispositional attribution (low ϕ_*Ext*_) renders character impressions more sensitive to others’ overt behavior. Combined with splitting, this could lead to rapid oscillation between extreme appraisals of others in situations where others’ behavior is changeable, in keeping with heightened interpersonal sensitivity. By comparison, a bias toward situational attribution (high ϕ_*Ext*_) tends to stabilize existing polarized impressions, and thereby encourages longer timescale relational and affective instability. Future work might examine how such attributional biases influence the stability of idealization and devaluation across environments with varying dynamics.

#### Negative Versus Positive Splits

Finally, within the split-HMM, a subject-specific parameter, ϕ_*Bad*_, governs the relative balance of *Bad*, as opposed to *Good* prior beliefs. In our analysis of moral inference data, we find that participants with BPD show more prominent negative prior beliefs, compared with non-BPD participants, captured in significantly higher estimates of ϕ_*Bad*_ among BPD participants. A possible interpretation is that this underlying pessimism about others’ character is a learned response to a hostile developmental environment. In support of this idea, people with BPD tend to recall more episodes of injury and negative experiences from their childhood when compared with non-BPD controls (e.g., Barnow et al., 2009; Nigg et al., 1992).

### Limitations and Future Directions

The model described here leaves open a number of avenues for future enquiry. Key questions for further research are as follows: (a) How are moral impressions revised? (b) In which contexts and along which dimensions does splitting occur? (c) Does splitting serve a defensive function? and (d) How might a split-HMM inform therapeutic interventions? We consider these in turn below.

#### Revising Moral Impressions

Extensive previous research shows that harmful actions are found to shape impressions of moral character to a greater extent than helpful actions, referred to as a “negativity bias” (Amabile & Glazebrook, 1982; Brown et al., 2005; Hamilton & Zanna, 1972; Rothbart & Park, 1986; for reviews, see Skowronski & Carlston, 1989; Ybarra, 2002). Thus, favorable impressions of another person are revised downward more readily than unfavorable impressions are revised upward (Briscoe et al., 1967; Reeder & Coovert, 1986; Siegel et al., 2018, Study 6). A widespread explanation for negativity bias is that negative behaviors are more diagnostic of underlying intent than are positive behaviors (Jones & Davis, 1965; Mende-Siedlecki et al., 2013; Reeder & Brewer, 1979; Reeder & Coovert, 1986; Skowronski & Carlston, 1989). Jones and Davis (1965) proposed that this might arise since positive actions can also serve ulterior, manipulative purposes. Ybarra (2002) advanced a subtler explanation, namely that, since helpful behavior is encouraged by societal norms while harmful behavior is discouraged, people are prone to infer that positive behaviors are caused by social demands, whereas negative behaviors are caused by dispositions (see also Reeder & Brewer, 1979; Vonk & Van Knippenberg, 1994). In all such accounts, negative behavior is seen as providing more information than positive behavior regarding underlying dispositions.

At first glance, a finding that healthy participants are faster to update their beliefs about “bad” as opposed to “good” others (Siegel et al., 2018, 2020) appears to contradict existing findings of a negativity bias. If healthy people quickly change their beliefs about bad others, ought they not readily forgive transgressions when behavior improves? Indeed, our model fitting results indicate that non-BPD participants are more likely than their BPD counterparts to engage *Integrated* representations of “bad” others, and therefore to reinstate positive impressions following ruptures. This effect is commensurate with experimental findings of slow recovery of cooperation in BPD following perceived defections (Hula et al., 2018; King-Casas et al., 2008).

However, a split-HMM allows for a possibility that this finding coexists with a degree of negativity bias. In keeping with the theories above, a split-HMM produces a negativity bias if the subject believes that situational factors can improve behavior (e.g., due to social pressures), but cannot worsen it (i.e., there are “no excuses”; Figure 7b). Importantly, when this arrangement is combined with an *Integrated* person prior (ϕ_*Split*_ = 0), subjects learn faster from unexpectedly bad behavior than from unexpectedly good behavior (illustrated in supplemental Figure S11). A split-HMM thus allows for the possibility that non-BPD participants are slower to revise impressions of “bad” people upward than to adjust their impressions further downward. We also find that both BPD and non-BPD participants exhibit a degree of idealization and are thereby slow to change their beliefs about “good” others in the face of minor misdemeanors. Taking these effects together, our model suggests that the average study participant is quick to forgive minor transgressions but slow to forgive major ones. Further empirical work might explore whether this prediction is indeed quantitatively consistent with how people revise moral impressions.

#### Domains and Dimensions of Splitting

Existing studies suggest that dichotomous thinking in BPD is more prominent in relational situations pertaining to themes of abandonment, abuse or neglect (Sieswerda et al., 2005; Veen & Arntz, 2000). In keeping with this idea, mentalizing ability is thought to deteriorate when emotions related to insecure attachment are provoked (Agrawal et al., 2004; A. Bateman & Fonagy, 2013; Choi-Kain et al., 2009; Fonagy & Bateman, 2008; Lyons-Ruth et al., 2005; Nolte et al., 2013, 2019). These ideas suggest that splits in BPD, rather than being domain-general, might pertain to particular forms of attachment relationship or relational schema; for example, “badness” might specifically correspond to feelings of abandonment, or “goodness” to an expectation of an all-fulfilling caregiver.

However, other research points to the possibility that splitting reflects a domain-general pattern of information processing. Furthermore, this pattern may not be limited to clinical groups. For example, reduced cognitive flexibility is associated with more polarized political judgments (Rollwage et al., 2018; Zmigrod et al., 2019, 2020; for reviews, see Rollwage et al., 2019; van Baar & FeldmanHall, 2022; Zmigrod, 2020), including biased updating of beliefs about the truth or falsity of political statements (Tappin et al., 2020). In a related sense, previous research has classified individuals according to their beliefs about the changeability of dispositions: “incremental” theorists believe that personality can develop and change, while “entity” theorists believe that personality traits are fixed (Dweck, 2008). These two theories can be seen as broadly corresponding to the integrated and split models considered here. Their validity is underlined by a relationship with real-world outcomes; for instance, people with an incremental theory are found to be more capable in recovering from failures (Hong et al., 1999). Taken together, such findings suggest that a tendency to perceive causal structure in terms of immutable categories might be a domain-general cognitive trait. Future work exploring this possibility and its manifestation, both in relational psychopathology and in the general population, could be of wide general interest.

##### Explicit Versus Implicit Social Inference

Here, we fitted a split-HMM to reported character judgments in response to explicit information about another person’s moral behavior. Importantly, existing studies have examined responses to *implicit* social information in BPD (e.g., Fineberg et al., 2018; Henco et al., 2020). For example, as described above Henco et al. (2020) studied a task in which one of two colored cards was more likely to be rewarded; a picture of a face, whose eyes were looking toward one of the two cards, provided additional probabilistic information about which card would be rewarded. However, participants were not instructed that eye gaze could be informative. Instead, participants were simply told that the face was included to make the experiment more interesting. Thus, the design probed peoples’ responses to implicit social cues that were either helpful or misleading.

Real social interactions require both explicit (slow/deliberative) and implicit (fast/automatic) processing. Previous authors have proposed that these functions are subserved by two systems for inferring others’ beliefs (Apperly & Butterfill, 2009), with partly distinct developmental and neural correlates (Frith & Frith, 2003; Van Overwalle & Vandekerckhove, 2013), a distinction which is also employed clinically in therapeutic approaches to personality disorder, such as mentalisation based therapy (Allen & Fonagy, 2006) and DBT (Linehan, 1993). A pressing direction for future research is therefore to compare the expression of splitting in implicit social learning paradigms with its manifestation in explicit character judgments.

Further research might also explore the extent to which “latent” priors, as modeled here, are accessible to self-report. For example, Siegel et al. (2018) considered that a possible explanation for why people are more uncertain about the moral character of bad, compared to good agents is a prior expectation that people behave morally, rendering the behavior of the bad agent more surprising. To examine this possibility, Siegel and colleagues collected data from a separate sample of participants, asking them how “most people would choose” for the choice options used in the task. Participants’ responses indicated an expected value of harm aversion (κ) that was not significantly different from 0.5; that is, equidistant between extremes of bad and good character. At first glance, this result appears to contradict our assertion of that non-BPD participants hold a prior that others can be extremely good. However, under our model, an extreme good representation for non-BPD participants itself has a low prior probability, and therefore may not be expected to be fully accessible to self-report before observing the good agent. The accessibility of representations as a function of their prior probability is an area for future study.

##### Unidimensional Versus Multidimensional Splitting

We have modeled splitting along a single axis of valence, from bad to good. Interestingly, however, previous studies (Napolitano & McKay, 2007; Sieswerda et al., 2005; Veen & Arntz, 2000) have found that people with BPD sometimes make extreme emotional evaluations of *opposite* valence toward the same character, for instance, rating someone as both highly reliable and highly jealous. Thus, in these studies, rather than classifying others as either “all-good” or “all-bad,” BPD patients displayed dichotomous thinking along a range of dimensions. Veen and Arntz (2000) concluded that rather than showing *unidimensional* dichotomous thinking, as implied by classical psychoanalytic accounts of splitting, BPD patients display *multidimensional* dichotomous thinking.

Veen and Arntz (2000) noted that the design of their study prompted BPD participants to rate various prespecified emotional dimensions and might therefore have tended to promote multidimensional emotional thinking. Indeed, a follow-up study with the same participant groups (Arntz & Veen, 2001) using an open-ended response format, found that BPD participants described the film characters as more affectively polarized and along fewer affective dimensions, when compared with controls, a pattern more consistent with classical accounts of splitting. Nevertheless, these findings draw attention to two important considerations: first, that splitting is not restricted to a single bad-good axis, and second, that collapsing emotional evaluations onto a single bad–good axis is itself an impoverished form of representation (see also Streufert & Streufert, 1969). Additional research might furnish these findings with a Bayesian interpretation in terms of the relative dimensionality of an internal state representation.

##### Splits in Self-Representation

As outlined in recent computational approaches (Smith, Kuplicki, et al., 2020; Smith, Lane, et al., 2019; Smith, Parr, et al., 2019), recognition of one’s own emotions can be conceptualized as Bayesian inference. Thus, a formally identical scheme to that outlined in the simulations above can be applied to inference about the self. Here, observations might consist of feedback regarding one’s own performance (*o*_1_) and external conditions (*o*_2_). Internal hidden states would then entail an appraisal of performance or self-esteem.

Applied to the self, an *Integrated* prior (“ego”) would tend to prevent extreme inference regarding one’s own internal state. By contrast, with a split self-representation, patterns of idealization and devaluation of the *self* would emerge. Based on our model, instances of good feedback could then engender extremely high self-esteem, with poor performance tending to be explained away (i.e., internal attribution of success and external attribution of failure; cf. Fitch, 1970; Miller & Ross, 1975; Nisbett & Ross, 1980; Ross, 1977; Zuckerman, 1979). However, inflated self-appraisals would be liable to collapse in the face of particularly bad feedback. Devaluation of the self would follow, at which point improvements would be explained away—preventing recovery in self-esteem (i.e., external attribution of success and internal attribution of failure).

It appears plausible that disrupted self-inferential processes of this kind might lead to both the “markedly and persistently unstable self-image or sense of self” and “intense and unstable emotions” that characterize BPD (American Psychiatric Association, 2013; Bender & Skodol, 2007; Kernberg, 1967, 1985; Koenigsberg, 2010). Self-idealization is also consistent with an inflated, grandiose sense of self observed chronically in narcissistic personalities (American Psychiatric Association, 2013; World Health Organization, 1992, 2018). More speculatively, splits in self-representation might also characterize mood disorders. For example, a split-HMM suggests, in general terms, that depression and associated negative attributions (Forgas et al., 1990; Rizley, 1978; Seligman et al., 1979) could arise from activation of latent devalued beliefs about the self (see Beck, 1963). This suggestion accords with a recent perspective that mood itself can be treated as a prior that shapes the perception of reward (Clark et al., 2018).

#### Defensive Splitting

According to the psychodynamic theory, splitting serves a defensive function, by preventing generalization of threat, and attributing sources of threat externally (through projection; Feldman, 1992; Hinshelwood, 1989; Kernberg, 1967, 1985; Klein, 1946; Kohut, 1966, 2013). Commensurate with this idea, a model of paranoia as “defensive avoidance” proposes that paranoid subjects defensively infer that they are under threat from others, to specifically avoid internalizing threats to their self-esteem (Bentall et al., 1991, 2001; Fornells-Ambrojo & Garety, 2009; though see Moutoussis et al., 2015; Murphy et al., 2018). Similarly, in cognitive and computational theories, optimistic or self-serving biases are often explained by postulating that thoughts and beliefs hold value in and of themselves (Bromberg-Martin & Sharot, 2020).

In the model proposed here, no such additional values are required. For instance, when splitting is applied to the self, a self-serving bias results simply from a prior expectation that the self is good, which ensures that evidence to the contrary is plausibly assigned to factors outside of the self, in a procedurally rational manner. It is necessary to postulate neither a specific self-aggrandizing agency that becomes active when in an elevated state, nor a specific self-punitive agency that becomes active when self-esteem is low. Thus, the model presented here accounts for aspects of splitting that appear defensive in a psychoanalytic sense, without postulating a specific defensive agency. Nevertheless, we note that a split-HMM might be adapted to account for defensive phenomena by assigning values or “goal-priors” to more desirable internal states (Friston et al., 2017). This could allow, for example, for idealization to arise as a compensatory response when faced with potential threat.

##### Splitting in Response to Uncertainty

A related idea is that splitting might occur defensively in response to *uncertainty*. Thus, a person might render their internal world more predictable by espousing either an idealized or a devalued view. Indeed, people with greater intolerance of uncertainty are more prone to form polarized views in response to political information (van Baar et al., 2021). Furthermore, a recent model based on similar methodology to the split-HMM, proposes that a preference for certainty explains the genesis of delusions (Erdmann & Mathys, 2022). Under this model, delusional subjects preferentially select high-precision explanations for their observations, resulting in a tendency to ascribe data to an increasing number of overly specific causes. This putative process accords with psychoanalytic notions of psychosis as entailing a “splintering” or fragmentation of mental content (e.g., Bion, 1957). The ethos of the aforementioned study is in keeping with the modeling approach we describe here, wherein subjects select hidden causes that minimize uncertainty about their observations, and where split causes are precise by definition. However, further research is needed to examine how individual differences in uncertainty tolerance might relate to splitting within the current modeling framework.

#### Therapeutic Applications of a Split-HMM

Finally, we propose that models such as the split-HMM, based on an evolution of latent states across time, have potential to generate novel psychotherapeutic interventions. First, in keeping with existing therapeutic approaches (e.g., DBT, CBT), a client might work together with a therapist to construct descriptive state-space models of how their thoughts, feelings and emotions, or those of others, evolve across time. For instance, a client might be asked to map out how qualities they idealize in another person change across time, and how these are balanced by less desirable qualities. The emphasis here would be on descriptively identifying split or unintegrated states, and their associated attributions. Relevant to this endeavor, we have shown that promoting contextualized appraisals of others can ameliorate milder degrees of splitting. Second, models of the form presented here could be fitted to clients’ responses, to derive behavioral measures of splitting or integration. This might be achieved, for instance, through simulated social interactions within a computational “task” designed to engage the relevant representations. The computational model advanced here permits identification of response styles associated with particular parameter settings (i.e., “computational phenotypes”; see, Montague et al., 2012). As a result, interactions can be created with real or simulated others who are preselected to respond in particular ways. For instance, by interacting with others who have differing degrees of splitting, participants might be helped to learn about their own social or affective responses. A key aim of such therapy would be to “loosen the grip” of excessively precise, extreme or one-dimensional representations of self, others and the world, and to foster more realistic and/or benign models. Such an approach would complement existing evidence-based therapies for BPD.

##### Using a Split-HMM as an Outcome Measure

Here, we cross-sectionally compared participants with BPD who had completed treatment in a DTC, and those who had not accessed such treatment. We found that a mean splitting parameter for the DTC group was lower than for the BPD group. The difference did not reach conventional levels of statistical significance (*p* = .075); however, this analysis was based on a small number of trials (34) per participant, with a between-subjects design. Future work might focus on assessing within-participant changes in splitting over the course of treatment, their relationship with symptom scores and functional improvement. For example, we hypothesize that successful DBT, which puts at its center overcoming all-or-nothing thinking by explicitly integrating alternative responses to a problem (Lynch et al., 2006), would result in a lessening of split priors.

### Conclusion

To conclude, we have introduced a model that parameterizes a tendency to make rigid category judgments about mental states as either “all-bad” or “all-good,” rather than flexible judgments along a continuous scale. Distinct from previous descriptive theories, the model makes quantitative predictions about how dichotomous beliefs emerge and are updated in light of new information. Specifically, the model addresses how dichotomous thinking is context-dependent, yet exhibits stability across time and is prone to abrupt changes of polarity. The model can also be fitted to empirical data, to measure individual susceptibility to relational and affective instability. We note that the model’s explanatory value will depend on its being adequately constrained in each respective scenario. However, the general framework proposed invites further work to study human relational phenomena across varying domains—and at different developmental stages—with potentially far-reaching implications.

## Supplementary Material

10.1037/rev0000430.supp

## Figures and Tables

**Table 1 tbl1:** States, Distributions, and Parameters of the Split-HMM

Variable	Interpretation	Process
Observations		
*o*_1_	Moral behavior	Observed
*o*_2_	Context cue	Observed
States		
*s*_1_	Internal state	Inferred
*s*_2_	External state	Inferred
*s*_3_	Person state	Inferred
Generative model		
$P\left(o_{1}|s_{1},s_{2}\right)$	Likelihood: behavior	Rigid
$P\left(o_{2}|s_{2}\right)$	Likelihood: context cues	Rigid
$P\left(s_{1,\tau }|s_{1,\tau -1}\right)$	Transition matrix: internal state	Rigid
$P\left(s_{2,\tau }|s_{2,\tau -1}\right)$	Transition matrix: external state	Rigid
$P\left(s_{1,\tau =0}|s_{3,t}\right)$	Dispositional prior	Rigid if *s*_3_ = *Bad* or *s*_3_ = *Good*, learned if *s*_3_ = *Integrated*
$P\left(s_{2,\tau =0}|s_{3,t}\right)$	Situational prior	Learned
*P*(*s*_3,*t*_)	Person prior	Learned
Parameters		
π_*o*1_	Likelihood precision: behavior	Free parameter
π_*o*2_	Likelihood precision: context	Fixed parameter
π_*s*1_	Precision of *Integrated* dispositional prior	Free parameter
ψ_*Ext*_	Weighting on nonneutral external states in situational prior	Free parameter
ψ_*Split*_	Weighting on *Bad* and *Good* relative to *Integrated* in person prior	Free parameter
ψ_*Bad*_	Weighting on *Bad* relative to *Good* in person prior	Free parameter
*Note*. HMM = hidden Markov model. Inference consists of inverting the model to estimate the hidden states likely to have generated observations at each timestep, τ, within a trial, *t*. Learning entails accruing information from trial-to-trial about which states a person is likely to occupy (via accumulation of concentration parameters in Dirichlet distributions).
