# Supplementary material for: A Social Inference Model of Idealization and Devaluation
Source: Psychol Rev. 2023 Aug 21;131(3):749–80. doi: 10.1037/rev0000430 (PMC11114086; doi:10.1037/rev0000430)
Supplement: Supplementary file 1 [file rev0000430_sm.docx]

**Supporting Material Online:**

**A Social Inference Model of Idealization and Devaluation**

Giles W. Story^1,2^, Ryan Smith^3,4^, Michael Moutoussis^5,2^, Isabel M. Berwian^6^, Tobias Nolte^5,7^, Edda Bilek^5^, and Raymond J. Dolan^2^

1 Division of Psychiatry, University College London

2 Max Planck-UCL Centre for Computational Psychiatry and Ageing Research, University College London

3 Laureate Institute for Brain Research

4 University of Tulsa

5 Wellcome Centre for Human Neuroimaging, University College London

6 Princeton Neuroscience Institute, Princeton University

7 Anna Freud National Centre for Children and Families

**Technical Specifications of a Split Hidden Markov Model**

We operationalize generative models following a previously published non-parametric Bayesian scheme (Friston et al., 2017; Smith, Parr, et al., 2019; Smith, Schwartenbeck, et al., 2020). Belief in hidden states is updated in light of observations according to Bayes’ rule. Letting $\boldsymbol{o}=\left\{ o_{1},\ldots,o_{n} \right\}$ denote the set of all observable random variables, and $\boldsymbol{s}=\left\{ s_{1}\ldots s_{f} \right\}$ denote the set of all hidden state factors, we can write:

$$P\left( \boldsymbol{s} | \boldsymbol{o} \right)=\frac{P\left( \boldsymbol{s} \right)P(\boldsymbol{o}|\boldsymbol{s})}{P(\boldsymbol{o})}$$

(S1)

The likelihood term, $P(\boldsymbol{o}|\boldsymbol{s})$, expressing statistical relationships between hidden states and data, can be further specified in terms of a probability distribution, with parameters $\boldsymbol{\theta}$.

**Inference: Inverting a Generative Model**

A Hidden Markov Model (HMM) extends the generative model to assume observations across a sequence of *T* time epochs, generated by an evolution of hidden states across time. For each hidden state factor at each level of the hierarchy, $l$ , a $K \times K$ transition matrix, $P(\boldsymbol{s}_{\tau}|\boldsymbol{s}_{\tau-1})$, determines the probability that each of$K$ hidden states will follow one another. A generative model (the numerator of Bayes’ rule) for a given level of the hierarchy can then be written as the joint distribution over observations, hidden states and parameters as follows:

$$P\left( \tilde{\boldsymbol{o}},\tilde{\boldsymbol{s}}\boldsymbol{,\theta} \right)=P(\boldsymbol{s}_{\boldsymbol{0}})P(\boldsymbol{\theta})\prod_{\tau=1}^{T} P\left( \boldsymbol{o}_{\tau} | \boldsymbol{s}_{\tau} \right)P(\boldsymbol{s}_{\tau}|\boldsymbol{s}_{\tau-1})$$

(S2)

Where$\tilde{\boldsymbol{o}}\boldsymbol{=}{\boldsymbol{\{o}}_{\boldsymbol{1}}\boldsymbol{\ldots}\boldsymbol{o}_{\boldsymbol{T}}\boldsymbol{\}}$ denotes the sequence of observations, $\tilde{\boldsymbol{s}}\boldsymbol{=}{\boldsymbol{\{s}}_{\boldsymbol{1}}\boldsymbol{\ldots}\boldsymbol{s}_{\boldsymbol{T}}\boldsymbol{\}}$ denotes the sequence of hidden states over time, and $\boldsymbol{\theta}$ denotes the model parameters. Note that $P(\boldsymbol{s}_{\boldsymbol{0}})$ is a prior over the starting states, while the transition matrix acts as a prior over states conditioned on the states at the previous time step.

We define a likelihood term, $P\left( \boldsymbol{o}_{\tau} | \boldsymbol{s}_{\tau} \right),$as a matrix, **A**. Each entry $A_{\boldsymbol{jk}}$ of this matrix represents the probability of given observation $o=j$ being generated when a particular setting of a given hidden state, $s=k$ is active. Specifically, for each hidden state factor at each time point, $P\left( o_{\tau} | s_{\tau} \right)$ is a multinomial likelihood distribution governing the probability of observing each outcome at each level of the hidden state. We generate the multinomial distribution at each level of hidden state from a discretized Gaussian distribution, with a specified mean and variance (see Figures 2 and 3).

We define a transition probability $P\left( \boldsymbol{s}_{\tau} | \boldsymbol{s}_{\tau-1} \right)$ as a matrix, ***B***. We specify this as a discrete random walk over different levels of each hidden state dimension. Unless otherwise specified, we configure the random walk with near-zero volatility, corresponding to a belief that internal states are unlikely to change within a given epoch. Finally, we define a prior distribution over starting states, $P\left( \boldsymbol{s}_{\boldsymbol{0}} \right)=$ $P\left( \boldsymbol{s}_{\boldsymbol{\tau=0}} \right)$, as a vector, *D*.

Using notation $Cat\left( \mathbf{A} \right)=Pr(o=j|s=k)$, we can write:

$$P\left( \boldsymbol{o}_{\tau} | \boldsymbol{s}_{\tau} \right)=Cat(\mathbf{A})$$

$$P\left( \boldsymbol{s}_{\tau} | \boldsymbol{s}_{\tau-1} \right)=Cat(\mathbf{B})$$

$$P(\boldsymbol{s}_{\boldsymbol{0}})=Cat(D)$$

(S3)

The starting configurations of **A**, **B** and *D* are given by Dirichlet distributions, the sufficient statistics, $\boldsymbol{\theta=\{}\mathbf{a}, \mathbf{b}, d\}$**,** of which are concentration parameters that can be regarded as the number of occurrences encountered in the past (Friston et al., 2017; Gershman & Blei, 2012).

HMMs are implemented according to the above scheme at both first and second levels. We denote Dirichlet distributions at each level with a subscript, for example the first-level likelihood matrix is written $\mathbf{A}_{1}$, indicating $\mathbf{A}_{l=1}$. Model inversion follows an iterative procedure, whereby a likelihood matrix at the second-level acts as a prior over starting states at the level below. At each first-level epoch, the current priors are used to infer first-level starting states, which then act as observations for inference at the second level.

***First-level Likelihoods***

As described in the Main Text and Figures 1 and 2, internal $\left( s_{1} \right)$and external hidden states $\left( s_{2} \right)$ jointly generate observed behavior,$o_{1}$. We operationalize observed behavior along an eleven-point ordinal scale from 0 to 1, representing the objective level of cost or benefit for the subject. The subject has a representation of $s_{1}$ with five states, corresponding to varying degrees of benevolence in another’s intentions: $s_{1}\boldsymbol{=}\{1=Bad, 2=Moderately Bad, 3=Neutral, 4=Moderately Good,5= Good\}$. If the generative model is run forwards, each $s_{1}$ emits behavior drawn from a Gaussian distribution:

$$P\left( o_{1}|s_{1}=x,s_{2}=Neutral \right)\mathcal{\sim N(}o_{1}; \mu_{x},\sigma^{2})$$

(S4)

Where:

$$\mu_{1}, \mu_{2}, \mu_{3}, \mu_{4},\mu_{5}=0, 0.25, 0.5, 0.75, 1$$

(S5)

And for all $x$:

$$\sigma^{2}=\frac{1}{\pi_{s1}}$$

(S6)

Thus, better intentions tend to generate better behaviour. For instance $s_{1}=2$entails that $o_{1}\sim N(\mu=0.25,1/\pi_{s1}$). For each $s_{1}$, Gaussian likelihoods are evaluated at the eleven possible settings of $o_{1}$, and normalized to sum to one, creating a discrete distribution.

The subject also has a representation of $s_{2}$ with five states. $s_{2}\boldsymbol{=}\{1=Very Unfavorable, 2=Unfavorable, 3=Neutral, 4=Favorable,5= Very Favorable\}$. If the model is run forwards, external states influence the expression of behavior by shifting the mode of the likelihood distribution:

$$P\left( o_{1}|s_{1}=x,s_{2}=y \right)\mathcal{\sim N}\left( o_{1}; \mu_{x}+\Delta_{y},\sigma^{2} \right)$$

(S7)

Where:

$$\Delta_{1}, \Delta_{2}, \Delta_{3}, \Delta_{4},\Delta_{5}=-0.5, -0.25, 0, 0.25, 0.5$$

(S8)

Thus, unfavorable external states worsen behaviour, by shifting the mode of the likelihood distribution towards poorer behaviour, while Favorable external states improve behavior, by shifting the distribution towards better behavior.

**Negative and Positive Bias in External Attribution.** To simulate a negativity bias, we set:

$$\Delta_{1}, \Delta_{2}, \Delta_{3}, \Delta_{4},\Delta_{5}=0, 0, 0, 0.4, 0.8$$

(S9)

Thus, favorable external states shift the likelihood distribution further towards better behaviour, increasing a capacity to infer ulterior motives, while unfavourable external states have no influence, thus removing a capacity to excuse bad behavior. To simulate a positivity bias, we set:

$$\Delta_{1}, \Delta_{2}, \Delta_{3}, \Delta_{4},\Delta_{5}=-0.8, -0.4, 0, 0, 0$$

(S10)

Thus, unfavorable external states shift the mode of the likelihood distribution further towards poorer behaviour, increasing a capacity to infer excuses, while favourable external states have no influence, removing a tendency to infer ulterior motives.

***Second-level Likelihoods***

At the first level, a prior over starting states ($D_{1}$) is specified by a likelihood matrix ($\mathbf{A}_{2}$) at the level above, with concentration parameters $\boldsymbol{a}_{2}$. At each higher-order time step, $t$, a ‘dispositional’ prior, $P\left( s_{1,\tau=0} | s_{3,t} \right)$, specifies a probability distribution over starting internal states, $s_{1,\tau=0}$, and a corresponding ‘situational’ prior, $P\left( s_{2,\tau=0} | s_{3,t} \right)$, specifies a probability distribution over starting external states, $s_{2,\tau=0}$.

**Dispositional Priors.** Dispositional priors are initialised for *Bad*, *Integrated* and *Good* person states, as described in the Main Text. We configure $P\left( s_{1} | s_{3}=Integrated \right)$ such that others’ internal state is expected to start as *Neutral* with some uncertainty around this estimate; specifically, internal states are drawn from a discretized Gaussian distribution:

$$P\left( s_{1,\tau=0} | s_{3,t}=Integrated \right)\mathcal{\sim N(}s_{1}; \mu, \sigma^{2})$$

(S11)

Where $\mu=3$ and $\sigma^{2}=1/\pi_{s1}$. The precision, $\pi_{s1}$, determines the extent to which *Integrated* dispositions constrain internal states at the level below. Split priors over dispositions, $s_{3}=\left\{ Bad, Good \right\},$prescribe a strong dominance of either *Bad* or *Good* internal states respectively. Split priors are configured such that:

$$P\left( s_{1,\tau=0} | s_{3,t}=Bad \right)\mathcal{\sim N(}s_{1}; 1, 1/3)$$

$$P\left( s_{1,\tau=0} | s_{3,t}=Good \right)\mathcal{\sim N(}s_{1}; 5, 1/3)$$

(S12)

For each $s_{3},$Gaussian likelihoods are evaluated at the five possible settings of $s_{1}$, and normalized to sum to one, creating a discrete distribution.

**Situational Priors.** Situational priors are initialised with a concentration parameter, $\varphi_{Ext}$, which governs the relative prominence of external states at the first-level. Specifically, the relevant dimensions of $\boldsymbol{a}_{2}$ are configured such that distributions over the five levels of first-level external states on the first trial are given by:

$$P\left( s_{2,\tau=0} | s_{3,t}=Bad \right)=\left\{ 0 0 1-\varphi_{Ext} \frac{\varphi_{Ext}}{2} \frac{\varphi_{Ext}}{2} \right\}$$

$$P\left( s_{2,\tau=0} | s_{3,t}=Integrated \right)=\left\{ \frac{\varphi_{Ext}}{4} \frac{\varphi_{Ext}}{4} 1-\varphi_{Ext} \frac{\varphi_{Ext}}{4} \frac{\varphi_{Ext}}{4} \right\}$$

$$P\left( s_{2,\tau=0} | s_{3,t}=Good \right)=\left\{ \frac{\varphi_{Ext}}{2} \frac{\varphi_{Ext}}{2} 1-\varphi_{Ext} 0 0 \right\}$$

(S13)

Thus $\varphi_{Ext}$, bounded between 0 and 1, controls the weighting on non-neutral relative to neutral external states. Setting $\psi_{Ext}=0$ denotes an expectation that $s_{2}\boldsymbol{=}\{Neutral\}$ for all $s_{3,t}$. Changes in observed behavior are therefore attributed to changes in internal state. By contrast, increasing values of $\psi_{Ext}$ encourage attribution to unfavorable or favorable external conditions. For $s_{3,t}=Bad$, $\psi_{Ext}>0$favorable external attributions are possible, embodying an expectation that bad people can behave well due to ulterior motives or situational pressures. For $s_{3,t}=Good$, $\psi_{Ext}>0$unfavorable external attributions are possible, embodying an expectation that good people can behave poorly in adverse contexts. (The key mechanism by which external attributions stabilize splitting is qualitatively unchanged by setting identical situational priors for all person states).

***Person Priors***

At the second level, $D_{2}$ represents a prior distribution over person states, ${P(s}_{3,t=0})$, the concentration parameters of which are initialised as:

$$d_{2}=\{\delta_{Bad}, \delta_{Integrated}, \delta_{Good}\}$$

(S14)

Where $\delta$ denotes the concentration parameter for each person representation on the first trial. We constrain this vector such that:

$$\delta_{Bad}=\psi_{Bad} \times\psi_{Split}$$

$$\delta_{Integrated}=1- \psi_{Split}$$

$$\delta_{Good}={(1-\psi}_{Bad}) \times\psi_{Split}$$

(S15)

And:

$$0\leq\varphi_{Bad}\leq1$$

$$0\leq\varphi_{Split}\leq1$$

(S16)

Setting $\psi_{Split}=0$ prevents splitting, while setting $\psi_{Split}=1$ allows only splitting. Similarly, setting $\psi_{Bad}=0$ turns off a Bad mode, while setting $\psi_{Bad}=1$ turns off a Good mode.

**Learning: Updating a Generative Model**

A series of first-level observations, $\tilde{\boldsymbol{o}}\boldsymbol{=}{\boldsymbol{\{o}}_{\boldsymbol{t=1}}\boldsymbol{\ldots}\boldsymbol{o}_{\boldsymbol{t=}\mathbf{T}}\boldsymbol{\}}$, is denoted as a *trial*, after which concentration parameters are updated to mediate learning. We configure models with a single second-level observation step per trial, i.e. $T=1$. On each *trial*, first-level observations $\tilde{\boldsymbol{o}}\boldsymbol{=}{\boldsymbol{\{o}}_{\boldsymbol{\tau=1}}\boldsymbol{\ldots}\boldsymbol{o}_{\boldsymbol{\tau=}\mathbf{T}}\boldsymbol{\}}$, are used to infer first-level starting states, ${\hat{\boldsymbol{s}}}_{\tau=0}$. These in turn provide observations, ${\hat{\boldsymbol{s}}}_{\tau=0}=\boldsymbol{o}_{\boldsymbol{t=}\mathbf{1}}$, to infer second-level states. At the end of each trial, *learning* proceeds by adding counts to the second-level concentration parameters $d_{2}$ and $\mathbf{a}_{\mathbf{2}}$, reflecting the frequency of inferred person states and their contingency with first-level states.

Concentration parameters of a likelihood distribution, $\mathbf{a}_{\mathbf{2}}$, are updated by accumulating coincidences between second-level and first-level states as follows:

$$\boldsymbol{a}_{trial+1}={\omega\boldsymbol{a}}_{trial}+ \eta\boldsymbol{o}_{t=1} \bigotimes\hat{s}_{3t=1}$$

(S17)

Here$\bigotimes$ denotes the outer product, $\hat{s}_{3t}$ denotes a posterior distribution over person states, and $\boldsymbol{o}_{t}$ denotes observations, corresponding to inferred first-level starting states. Parameters $\omega$ and $\eta$ control the rate of updating: eta (η) is a learning rate, which controls how much inferred hidden states update concentration parameters after each trial, while omega (ω) is a forgetting rate, which controls the extent to which inference in recent trials overwrites previous updates. The above encompasses learning both dispositional and situational priors, generating internal and external state outcomes respectively. Learning over split dispositions, $s_{3}=\left\{ Bad, Good \right\}$, is prevented by multiplying the concentration parameters for the respective person-internal state mappings by a large number, such that new observations have little effect. Disposition learning therefore takes place only for $s_{3}=\left\{ Integrated \right\}$.

Concentration parameters,$d_{2}$, of a prior distribution over person states are updated as follows (in notation omitting the second-level subscript):

$$d_{trial+1}={\omega d}_{trial}+{\eta\hat{s}}_{3t=1}$$

(S18)

Where $\hat{s}_{3t=1}$ denotes a posterior belief over person states on the current trial. We fix forgetting rates and learning rates throughout all applications of the model considered here, setting $\omega=0.75$,$\eta=0.25$ when updating *d* and $\omega=0.8$,$\eta=0.2$ when updating **a**. Changes in beliefs are then governed by the relative precision of priors and likelihoods at each level.

**Model Fitting Procedures**

**First-level Model Specification for a Split-HMM**

To fit the model to moral inference data from the study of Siegel et al. (2020), we assume subjects have an accurate perceptual model of how an agent’s harm aversion influences their behaviour. Specifically, the subjective value to an observed agent of choosing the more harmful of the two options is given by:

$$V_{harm}\left( \kappa\right)=\left( 1-\kappa\right)\Delta_{m}-\kappa\Delta_{s}$$

(S19)

Where $\Delta_{m}$ and $\Delta_{s}$ respectively represent the difference in money (for the agent) and shocks (for a third party) between the two choice options (see, Siegel et al., 2018). At low $\kappa,$ the value of choosing the more harmful option depends more steeply on the amount of money gained for the agent. At high $\kappa,$ the (dis)value of choosing the harmful option depends more steeply on the level of shocks incurred for the third-party. Values are transformed into probabilities of the observed agent’s choosing the more harmful option on each trial by means of a softmax function:

$$P\left( o_{1}=harm | \kappa\right)=\frac{1}{1+e^{-\beta*V_{harm}\left( \kappa\right)}}$$

(S20)

The inverse temperature parameter, $\beta$, is set to 100, such that the function is close to deterministic; in other words, given a sampled value of $\kappa$, the simulated agent reliably chooses their preferred option.

We incorporate noise into the subject’s perceptual model by assuming that $\kappa$ on each trial is drawn from a Gaussian likelihood distribution. Specifically, we let internal hidden states, $s_{1}$, correspond to beliefs about the agent’s expected harm aversion, $\mu=E[\kappa$], ranging from 0 to 1 in increments of 0.1. The subject therefore has a representation of $s_{1}$ with eleven states. If the generative model is run forwards, each $s_{1}$ emits a setting of $\kappa$, drawn from a Gaussian distribution:

$$P\left( \kappa|s_{1}=x,s_{2}=Neutral \right)\mathcal{\sim N(}\kappa; \mu_{x},\sigma^{2})$$

(S21)

Where:

$$\mu_{1}, \mu_{2}, \mu_{3}\ldots\mu_{11}=0, 0.1, 0.2,\ldots1$$

(S22)

And for all $x$:

$$\sigma^{2}=\frac{1}{\pi_{s1}}$$

(S23)

For instance $s_{1}=2$ entails that $\kappa\sim N(\mu=0.1,\sigma^{2}=1/\pi_{s1}$). (Gaussian likelihoods are truncated such that $0<\kappa<1).$In other words, an agent’s choices provide noisy estimates of their underlying propensity to harm. This arrangement corresponds to the first-level likelihoods shown in Figure 2; whereas in Figure 2 internal states generate behaviour on an ordinal scale, here internal states generate a setting of $\kappa$, which is in turn transformed deterministically into a binary choice.

A likelihood function describing a probability of choosing the harmful option on each trial is obtained by marginalizing over emitted $\kappa$:

$$P\left( o_{1}=harm|s_{1},s_{2} \right)=\sum_{\kappa} P\left( o_{1}=harm|\kappa\right)P(\kappa|s_{1},s_{2})$$

(S24)

A subject’s belief about the agent’s harm aversion is represented by beliefs over internal states, $s_{1}$. Observing harmful choices increases a posterior probability for lower settings of harm aversion, while observing helpful choices increases a posterior probability for higher settings of harm aversion.

**Learning Rate Computation**

To permit comparison with previous studies, we summarise the rate of belief updating as an overall learning rate, $\alpha$. To do so we leverage a definition of learning rate based on a simple Rescorla-Wagner model, wherein:

$$\hat{o}_{\tau+1} = \hat{o}_{\tau} + \alpha(o_{\tau}- \hat{o}_{\tau})$$

(S25)

Here, outcome predictions, $\hat{o}_{\tau}$, are updated proportional to a prediction error, i.e. the difference between observed and predicted outcomes. By re-arranging the above equation, the learning rate $\alpha$ can be expressed as the slope of a relationship between belief updates and prediction errors across observations. Accordingly, we estimate $\alpha$ for a given parameterization of the model by regressing changes in predicted outcomes ($\hat{o}_{\tau+1}-\hat{o}_{\tau})$ against prediction errors $(o_{\tau}- \hat{o}_{\tau})$. Here, $\hat{o}_{\tau}$ denotes the maximum *a posteriori* (MAP) outcome given beliefs over hidden states *at the time of cue presentation* (i.e. before observing behaviour at the current time step). Note that $\alpha$ is an heuristic summary measure, which incorporates belief updating both within and between trials, and is distinct from the parameters used to mediate learning Dirichlet distributions, which are fixed throughout.

For simulations, observed behaviour, $o_{\tau},$ is on an ordinal scale. When estimating $\alpha$ we use MAP behaviour predicted by the model at cue time on each epoch as $\hat{o}_{\tau}$. For a model fitted to moral inference data, observed behavior is binary. We therefore estimate $\alpha$ for updates in expected $\kappa$. Here observed outcomes are given by the objective expected $\kappa$ implied by each of the agent’s choices taken individually. Predicted outcomes are the expected values of $\kappa$ implied by a subject’s MAP prediction of $s_{1}$ before observing behavior.

**Specification of a Hierarchical Gaussian Filter (HGF) Model**

Siegel et al. (2020) fitted participants’ choice predictions using a Bayesian model with an adaptive learning rate, the Hierarchical Gaussian Filter (HGF) (Mathys et al., 2011). For comparison, we also fit an HGF model to *moral character ratings*, following the general procedure described by Siegel et al. (2020). An HGF is similar in principle to the hierarchical HMM model describe above, in so far it postulates that a subject uses a generative model to infer the hidden states underlying their observations. A key difference is that, in the HGF, hidden states evolve on a continuous scale. Specifically, hidden states are assumed to evolve according to Gaussian random walks, the step-size of which is determined by a *volatility* parameter. A variational Bayesian approach is used to invert the model, leading to a set of closed-form update equations. A basic feature of the model is a division into a perceptual model, which describes how participants update their beliefs about hidden states from inputs, and a response models which describes how beliefs are used to make predictions.

Conventionally, hidden states under the HGF are referred to as $x$, where $x_{1}$ denotes a hidden state corresponding to the observations themselves. Siegel et al. (2020) considered two levels of hidden states, $x_{1}$, denoting the agent’s choices, and $x_{2},$ representing the agent’s harm aversion. Here we use a notation as introduced previously for HMMs, where $o_{1}$ denotes observations and $s_{1}$ a hidden state representing harm aversion. The probability that an agent will choose the less harmful option $o_{1}=1$, versus the harmful option, $o_{1}=0$, is governed by $s_{1},$which here is a continuous state evolving over time as a Gaussian random walk, and signifies belief about the agent’s propensity to choose the less harmful option.

The conditional probability of an agent’s choices, given $s_{1}$, is described by a Bernoulli function:

$$P\left( o_{1} | s_{1} \right)={\sigma\left( s_{1} \right)}^{o_{1}}{(1-\sigma\left( s_{1} \right))}^{1-o_{1}}$$

(S26)

Where $\sigma(\cdot)$ is a softmax function:

$$\sigma(x)≝\frac{1}{1+e^{-x}}$$

(S27)

The temporal evolution of $s_{1}$is governed by a participant-specific volatility parameter ω, which allows for inter-individual differences in learning rate. Given ω, and $s_{1}$at the previous time step ($\tau-1)$ a generative model for $s_{1}$ at the current time step, $\tau$, is given by:

$$P\left( s_{1,\tau} | s_{1,\tau-1}, \omega\right)\mathcal{=N(}s_{1,\tau}; s_{1,\tau-1},\exp\left( \omega\right))$$

(S28)

Variational Bayesian inversion yields an update equation under a

mean-field approximation, where beliefs are updated as a function of precision-weighted

prediction errors (see Mathys et al., 2011, for details). Within this update equation, prediction errors are given a larger weight when the precision of the belief about the agent’s preference (i.e. $\kappa$) is low, which arises when the log volatility, $\omega$, is higher. In summary, the HGF is equivalent to the first-level model of HMM models described above, but where $s_{1}$ (a latent state governing an expectation over harm aversion) evolves continuously, and where learning rate depends on volatility in $s_{1}$. The HGF contains no external state dimension and no higher-order dimension.

We fit the HGF to participants’ moral character ratings, using a decision model with a discretized Gaussian likelihood, mapping from beliefs about $s_{1}$ to reported ratings. The noise of this discretized Gaussian is fitted as a free parameter, $\varepsilon$. To allow comparison between HGF and HMM models, we discretize both $s_{1}$ and observed ratings into the same eleven bins used for fitting HMMs. We configure the HGF with priors as specified by Siegel et al. (2020). Following Siegel et al. the prior mean of$s_{1}$ is set to zero, resulting in a 0.5 probability that the agent will choose the more harmful option. We freely fit volatility and decision-noise parameters to character ratings for ‘bad’ and ‘good’ agents separately, resulting in four free parameters.

**Supporting Simulations**

**Idealization following Activation of a Good Latent Prior**

To simulate idealization, we implement a latent ‘Good’ person state, setting the prior probability of a ‘Bad’ person to zero ($\psi_{Bad}=0)$. With a solely integrated prior ($\psi_{Split}$=0), the subject tracks changes in behavior (Figure S3a). With a low prior probability of ‘Good’ ($\psi_{Split}$=0.075, Figure S3b), as behaviour improves, the subject switches to inferring that they are dealing with a Good person. A switch to idealization is faster when a Good person has higher prior probability ($\psi_{Split}$=0.25, Figure S3c). When behavior subsequently deteriorates, predictions remain optimistic. Here, idealization is stabilized by attributing less-than-perfect behaviour to Unfavorable external conditions. In other words, a person’s external circumstances are used to *excuse* behaviour that does not accord with expectations, leaving their perceived good intent untarnished. As shown previously for devaluation in the Main Text, attribution errors and learning effects combine to strengthen idealization over time, even in the face of countervailing evidence. These findings accord with observations that idealized others can ‘do no harm’ in the eyes of the idealizing subject.

**Supporting Figures**

**Figure S1**

*Simulations with an Integrated Dispositional Prior: Dispositional and Situational Learning*

**
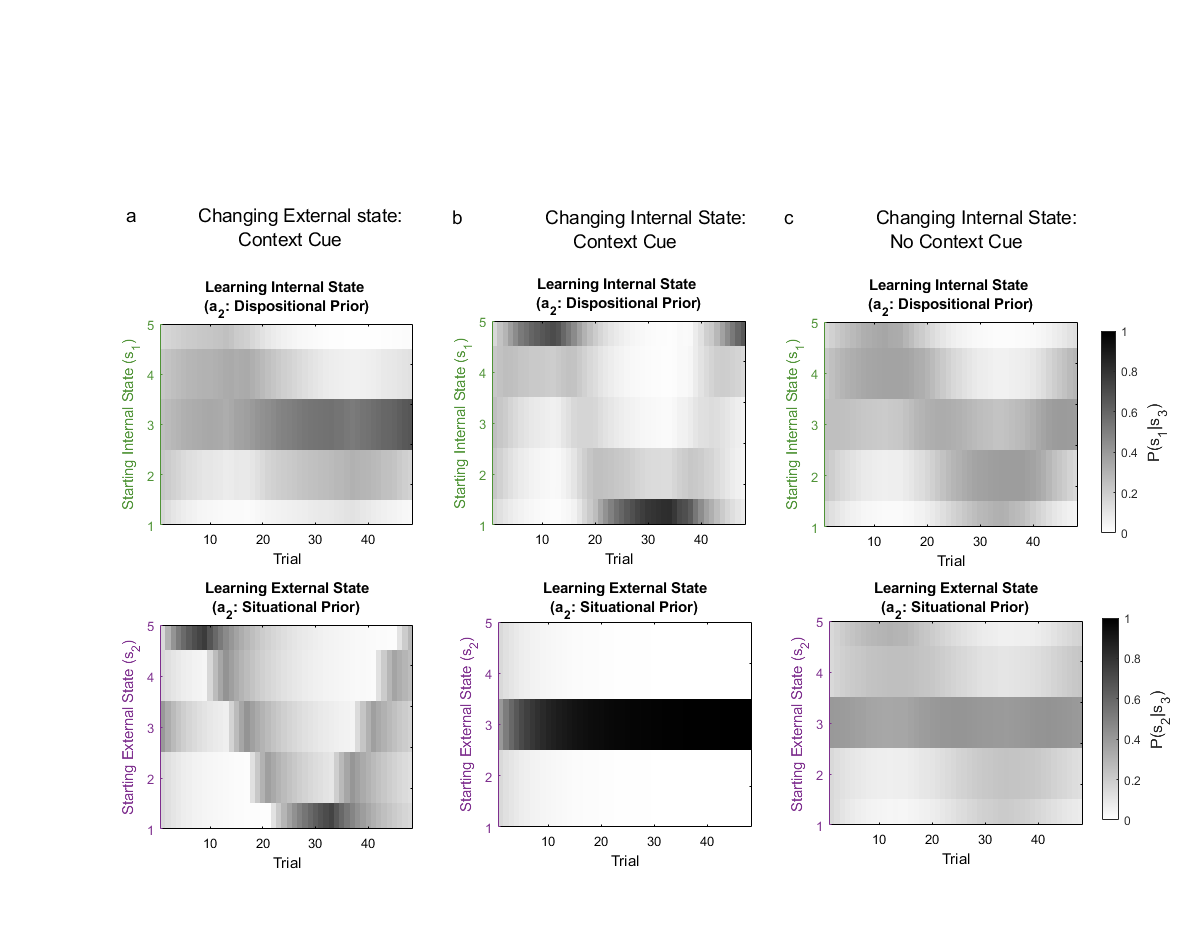
**

*Note*. Updates of dispositional and situational priors for the simulations shown in Figure 4. a) The subject observes a cue ($o_{2}$) providing reliable information ($\pi_{o2}= 2$) about external state changes. Observed behaviour (blue circles) gradually worsens, before improving again. The subject learns that external state is changing. b) Changing behaviour arises from changes in internal state. A cue ($o_{2}$) provides reliable information ($\pi_{o2}= 2$) that external state does not change. The subject accordingly updates a dispositional prior. c) Without a reliable external state cue, the subject partly attributes internal state changes to external factors, updating both situational and dispositional priors.

**Figure S2**

*Simulations with a Latent Bad Dispositional Prior: Dispositional and Situational Learning*

**
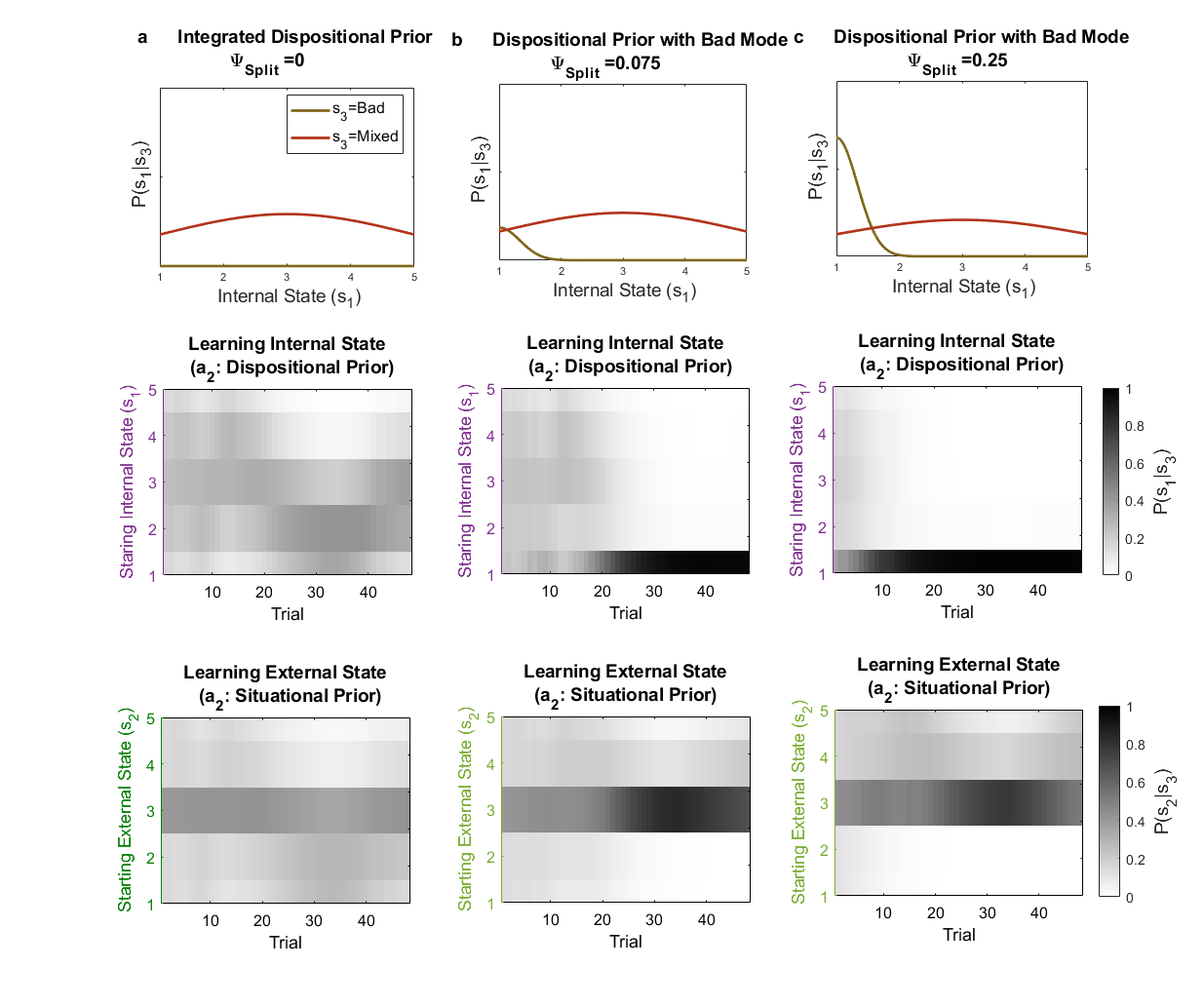
**

*Note*. Updates of dispositional and situational priors for the simulations shown in Figure 5. Top row: smoothed distributions representing the starting configuration of dispositional priors. Second row: matrices showing dispositional priors across time, averaged across expectations over *Bad* and *Integrated* representations. Third row: matrices showing situational priors across time. Column a) Inference with an integrated (unimodal) prior over internal states b) Inference with a latent prior that others are ‘all-bad’. Note that ambiguous information consolidates belief in a *Bad* person across time (middle panel, trial 15 onwards). c) A *Bad* latent prior with higher prior probability: the subject switches more readily to infer the other is ‘all-bad’.

**Figure S3**

*Inference with a Latent ‘Good’ Dispositional Prior: Idealization*


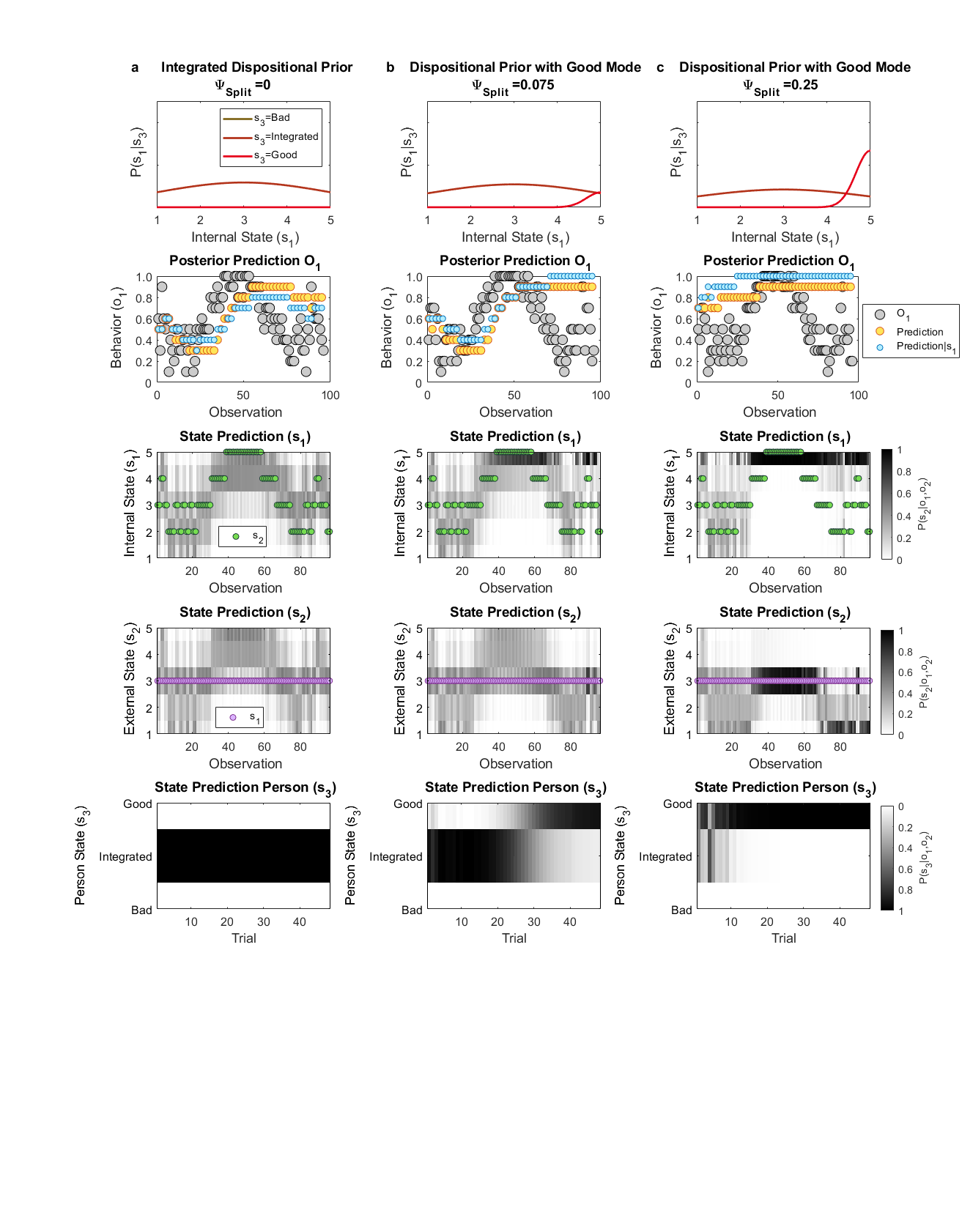


*Note*. Simulated data are plotted as in Figure 4 ($\pi_{s1}=0.5, \pi_{o2}=0.001$, $\pi_{o1}=0.25$). a) Inference with an integrated (unimodal) prior over internal states. b) Inference with a latent prior that others are ‘all-good’: after observing benevolent behavior the subject infers the other is a ‘Good’ person (fourth row, from trial 25 onwards), after which predictions remain optimistic; worsening behavior is then attributed to an unfavourable external factor (i.e. an ‘excuse’). As a result, ambiguous information consolidates belief in ‘Good’ person across time. (c) When a Good latent prior has higher prior probability the subject switches more readily to infer the other is extremely good.

**Figure S4**

*Higher Degrees of Splitting Distort Contextual Information*


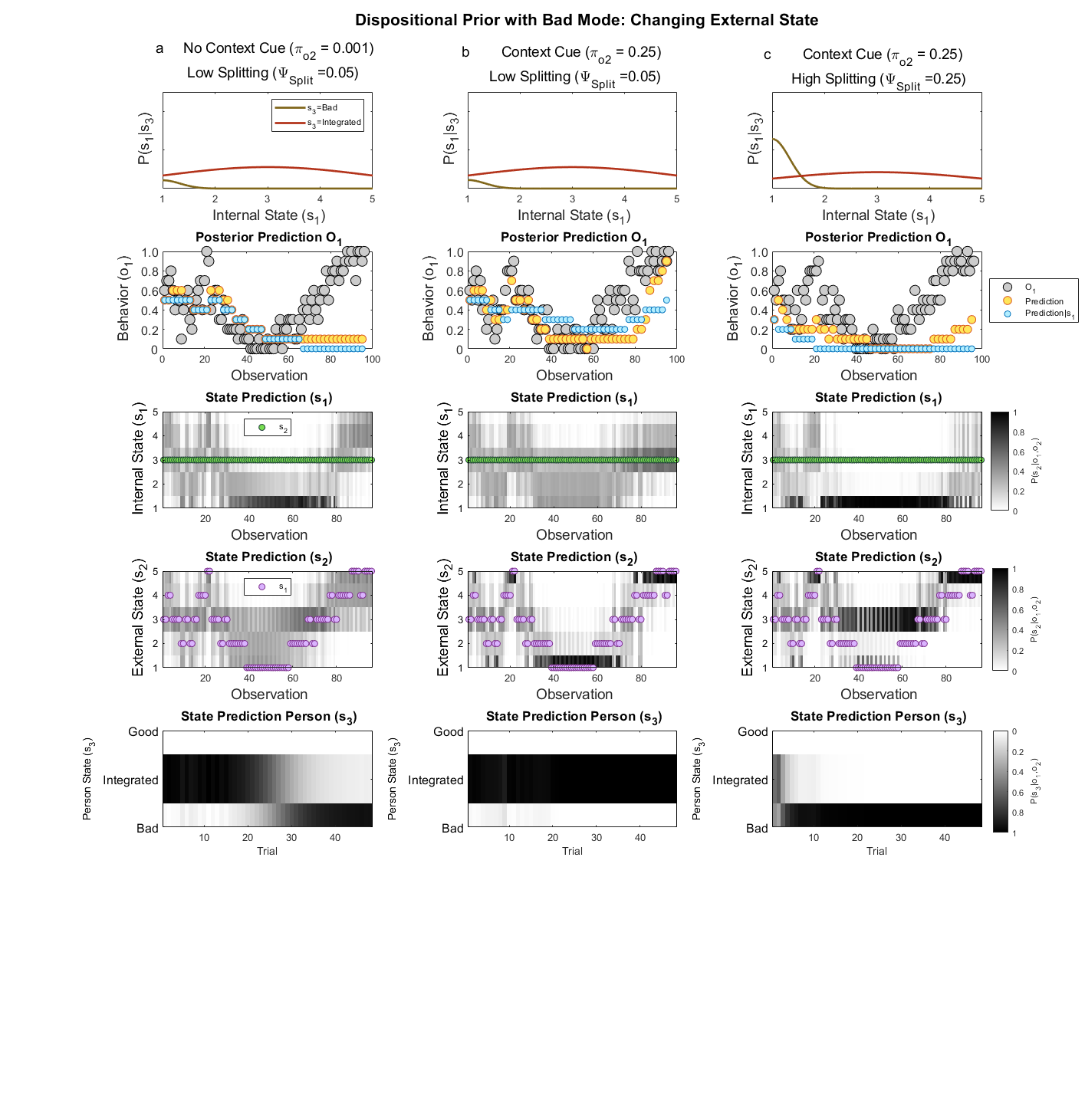


*Note*. Simulated data are plotted as in Figure 5b ($\varphi_{Bad}=1, \pi_{s1}= 0.5$, $\pi_{o1}= 0.25$), for varying cue precision, $\pi_{o2}.$ However, here the other’s *external* state (third row, purple circles) changes over time. ***Column a):*** Inference with a latent prior that others are ‘all-bad’: after observing poor behavior the subject incorrectly attributes their behavior to their being a *Bad* person (fourth row, from trial 15 onwards). In the absence of informative external state cues, improved behavior is attributed to a favorable external factor (or ‘ulterior motive’). ***Column b):***.Reliable external state information prevents devaluation. ***Column c):*** At higher levels of splitting, devaluation still occurs despite reliable external state information. Here, inference that the other is a *Bad* person distorts external state perception. This arises due to an interaction in the person’s generative model, such that *Bad* people can have ‘no excuses’.

**Figure S5**

*Latent Splitting with Modifiable Split Priors*


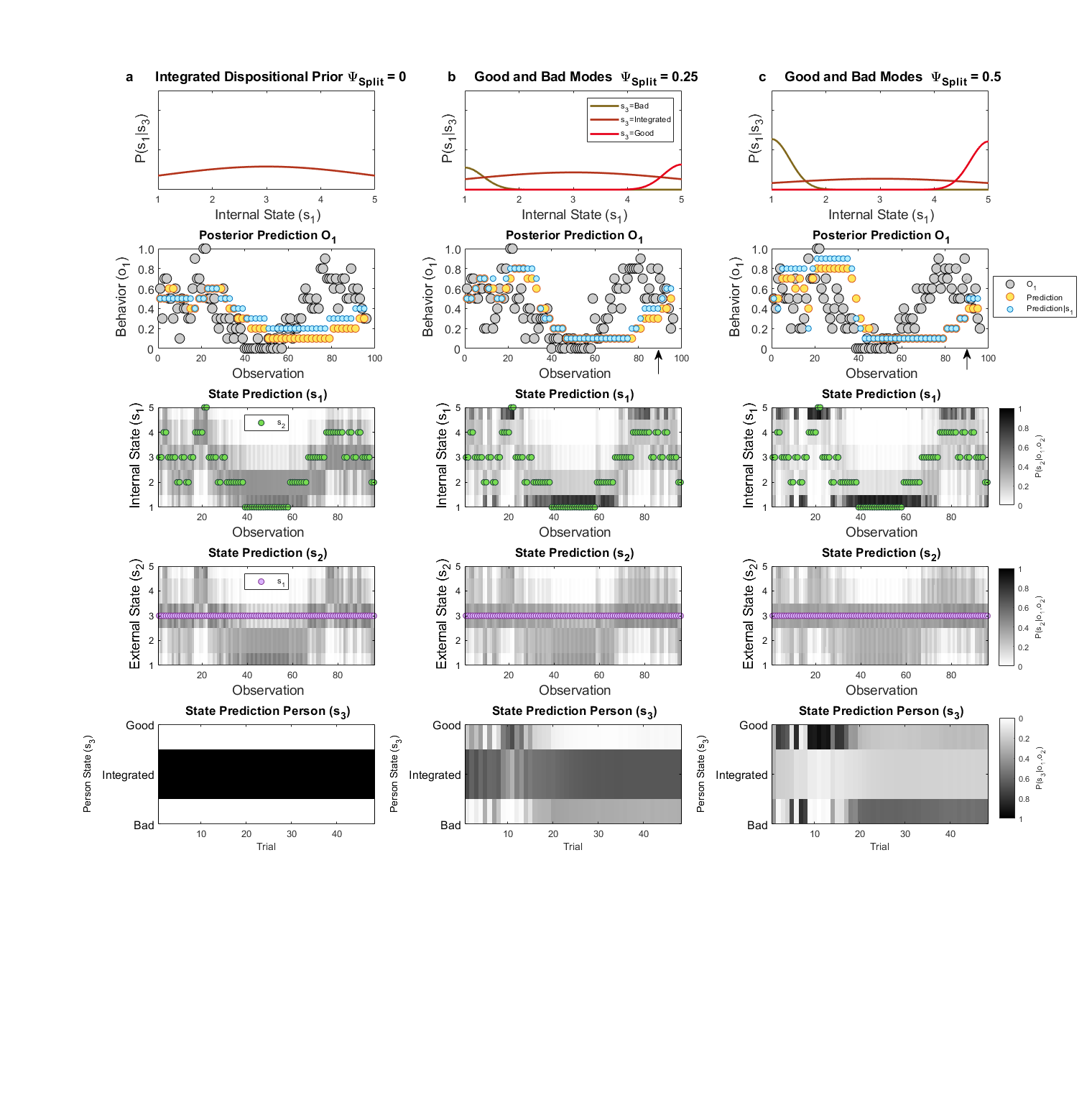


*Note*. Simulated data are plotted as in Figure 6 ($\pi_{s1}=0.5, \pi_{o2}=0.001$, $\pi_{o1}=0.25$), for varying degrees of splitting. However, here split priors can be modified through learning. ***Column a):*** Inference with an *Integrated* prior. ***Columns b)*** and ***c):***.Latent split priors ($\varphi_{Bad}=0.5)$. Here, phases of idealization and devaluation still occur, but become less marked across time as split priors begin to extinguish. In particular, by comparison with Figure 6c, at $\varphi_{Split}=0.25$, recovery from devaluation is possible (from observation 80 onwards, indicated by an arrow in row 2).

**Figure S6**

*Latent Splitting with Modifiable Split Priors: Dispositional Learning*

*
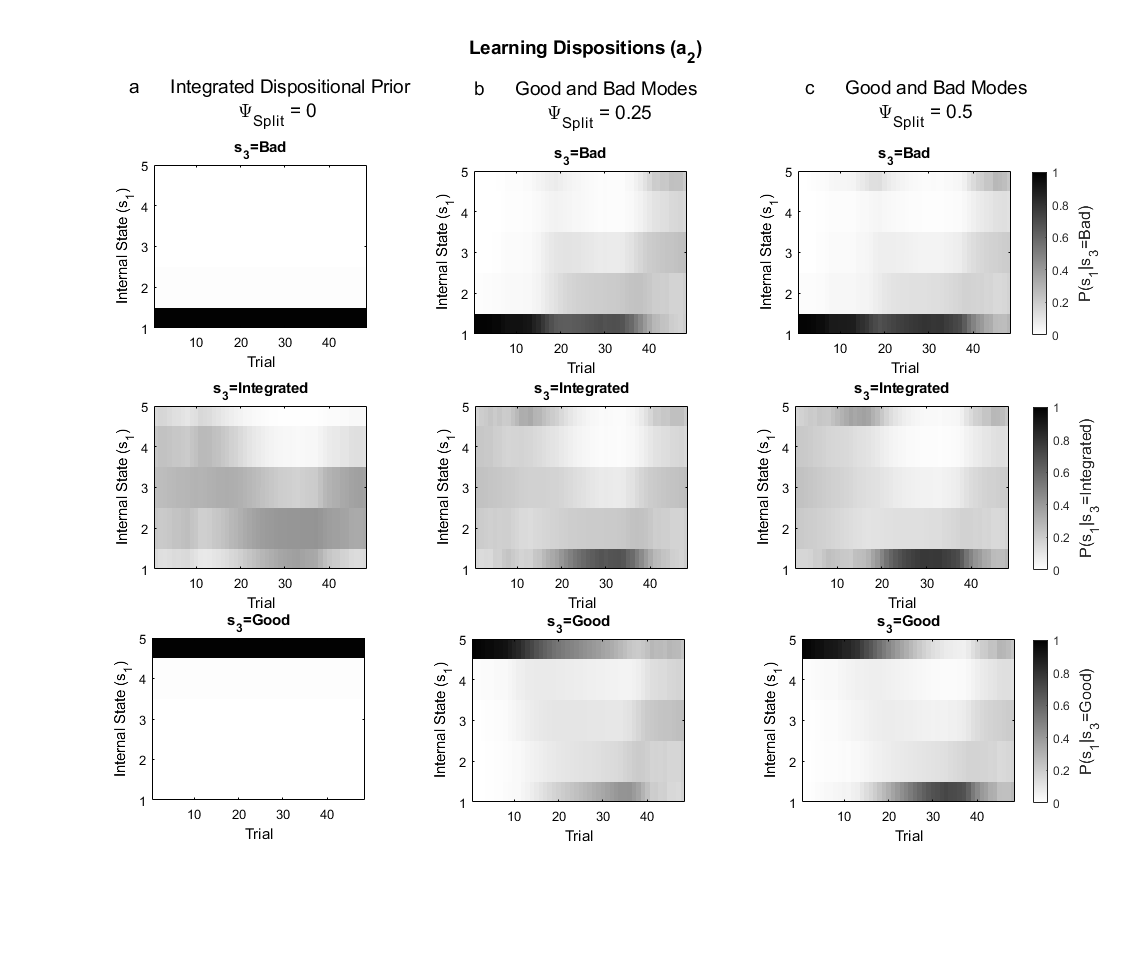
*

*Note*. Updates of dispositional priors for the simulations shown in Figure S5, shown for *Bad* (top row), *Integrated* (middle row) and *Good* (bottom row) dispositional priors across time. Column a) Inference with an *Integrated* prior over internal states. Columns b) and c) Inference with latent split priors. Although a degree of polarization remains, *Bad* and *Good* priors gradually lose their specific meaning across time. For example, a *Good* prior can itself become bimodal. This occurs firstly due to the way Dirichlet distributions are updated (simply by accumulating counts, rather than shifting the mean of a distribution), and secondly since person priors can ‘train’ each other. For example, a 70% belief that the other is a *Bad* person, and a 30% belief they are *Good*, may lead the subject to infer predominantly bad intentions, which become the data for updating both *Bad* and *Good* person priors, in proportion to their respective likelihoods. Thus a formerly ‘*Good*’ person prior may come to entail an expectation of bad intentions.

**Figure S7**

*Differential Learning from Good and Bad Observations at Varying Degrees of Splitting*


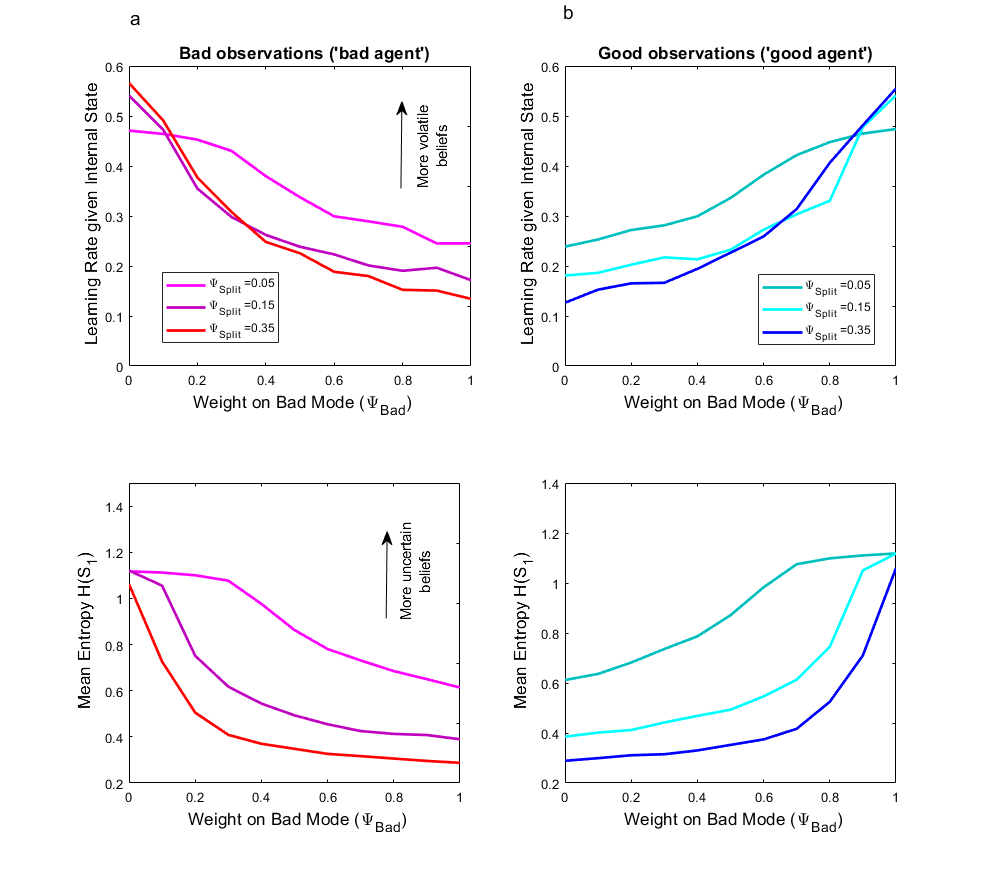


*Note*. Net learning rate and internal state uncertainty of a model with latent splitting for observations generated from ‘bad’ and ‘good’ agents, as shown in Figure 9 ( $\pi_{o2}= 0.001$, $\pi_{o1}= 0.25$, $\pi_{s1}= 0.5$, $\psi_{Ext}$=0.6), plotted here at varying settings of$\psi_{Bad}$ and $\psi_{Split}$. Column a) Learning rates and uncertainty over internal state for a ‘bad’ agent decrease, as the prior probability of a latent Bad mode, $\psi_{Bad},$ increases. Column b) Learning rate and uncertainty over internal state increase for a ‘good’ agent as the prior probability of a latent Bad mode, $\psi_{Bad},$ increases. These relationships are more convex at higher settings of $\psi_{Split}$. In summary, a more prominent Bad latent mode, $(\psi_{Bad}>0.5)$ entrains rigid, pessimistic beliefs in response to bad observations, while a more prominent Good latent mode, ${(\psi}_{Bad}<0.5)$entrains rigid, optimistic beliefs in response to good observations.

**Figure S8**

*Posterior Moral Character Ratings and Model Fits of an HGF Model*

*
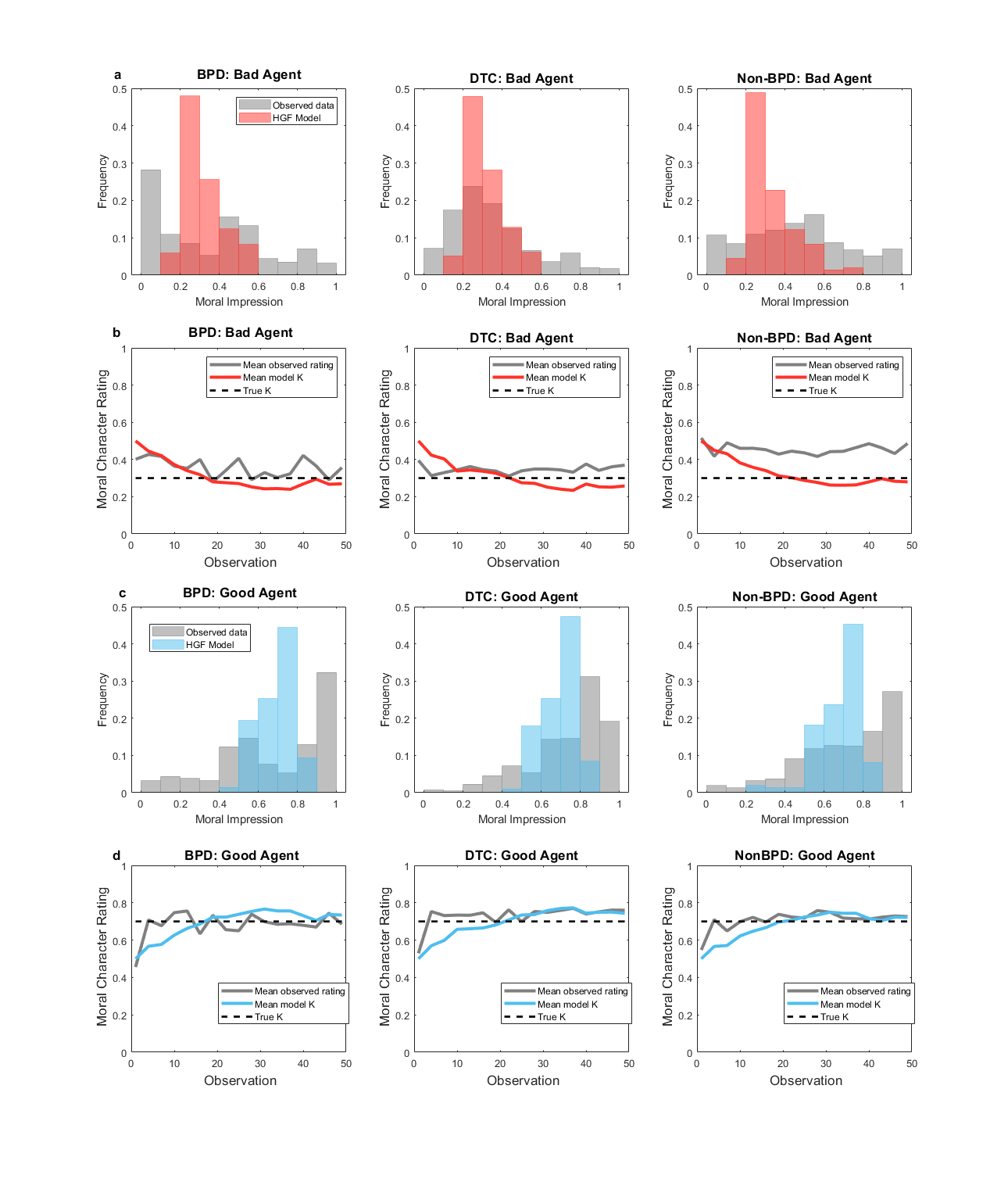
*

*Note.* a) Histograms showing distributions of posterior moral character ratings of the ‘bad’ agent across all trials for each group of participants. An Hierarchical Gaussian Filter (HGF) model approximates these with a unimodal posterior. b) Mean character ratings of the ‘bad’ agent across observations. Ratings of the ‘bad’ agent made by Non-BPD participants are optimistic relative to the true $\kappa$, however this is not captured within the HGF model c) Distributions of posterior moral character ratings of the ‘good’ agent across all trials for each group of participants. b) Mean character ratings of the ‘good’ agent across observations.

**Figure S9**

*Parameter Recovery of a Split-HMM*


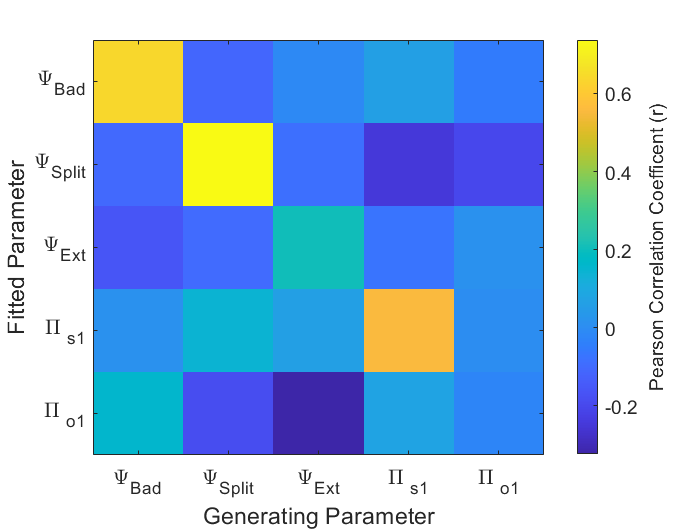


*Note*. Correlations between generating parameters and parameters extracted from fitting the Split-HM model to simulated data. 100 sets of moral character ratings were simulated from a Split-HMM with generating parameter values drawn from a uniform distribution between the bounds. Observations were the behaviour of ‘bad’ and ‘good’ agents shown to the first 100 participants in the study of Siegel et al. (2020). A Split-HMM was then fitted these datasets, to yield maximum likelihood parameter estimates. Parameter recovery for the key parameters indexing splitting ($\varphi_{Bad}, \varphi_{Split})$ is good, given only 34 trials per dataset. Poor parameter recovery for a propensity to attribute observations to external causes ($\varphi_{Ext})$, and first level precision ($\pi_{o1})$ are not unexpected, since the experimental design is not optimized to measure these parameters.

**Figure S10**

*Distribution of Model Parameters from Split-HMM Model Fits*


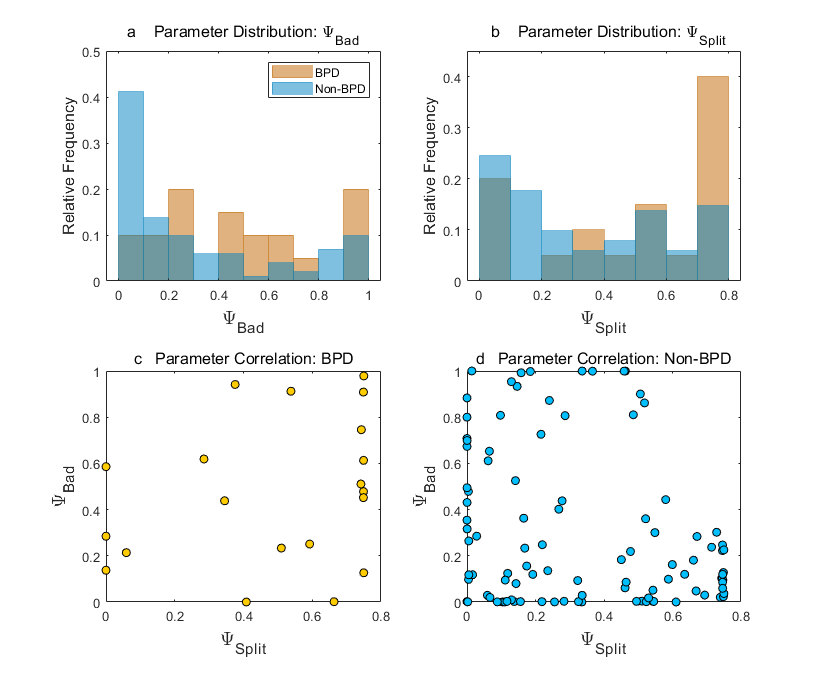


*Note*. a) Distribution of maximum likelihood estimates of $\psi_{Bad}$ across BPD and non-BPD participants. b) Distribution of maximum likelihood estimates of $\psi_{Split}$ across BPD and non-BPD participants. c) Scatterplot showing relationship between $\psi_{Split}$ and $\psi_{Bad}$ across BPD participants. d) Scatterplot showing relationship between $\psi_{Split}$ and $\psi_{Bad}$ across non-BPD participants. Few estimates fall in the top right-hand quadrant of the plot, indicating that high settings of both parameters distinguish BPD from non-BPD participants. Note that $\psi_{Bad}$ is undefined when $\psi_{Split}=0$, and in this event $\psi_{Bad}$ is expected to follow a uniform distribution between 0 and 1; this occurred in 15/145 participants.

**Figure S11**

*Learning Rate from Positive and Negative Prediction Errors: Integrated Dispositional Prior*


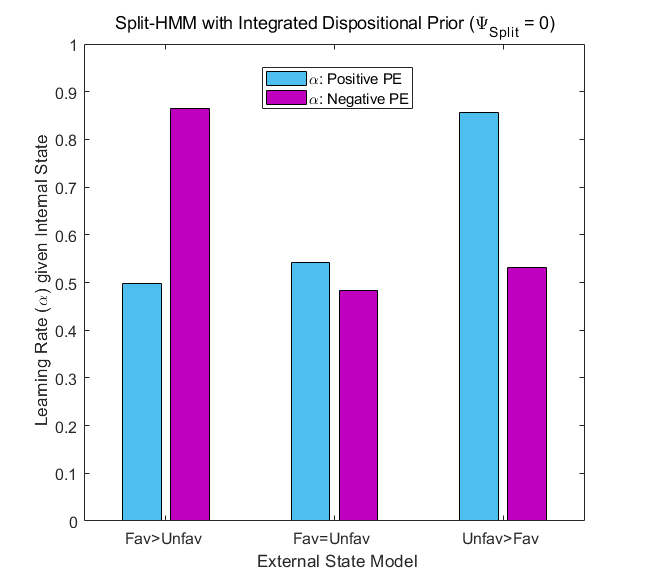


*Note*. Learning rates from positive and negative internal state ${(s}_{1})$prediction errors are shown for generative models with different configurations of the external state factor ${(s}_{2})$, with an Integrated dispositional prior ($\psi_{Split}=0, \psi_{Ext}=0.6, \pi_{o1}=0.25, \pi_{o2}=0.001, \pi_{s1}=0.5)$. In each case learning rates are derived from simulated responses to 100 sets of 96 observations sampled from an agent whose internal state starts as *Neutral*, and evolves on a random walk with a low level of volatility. Fav>Unfav entails an expectation that Favorable external states can improve behavior (e.g. an ulterior motive), while Unfavorable external states have no effect (i.e. no excuses); here subjects are faster to revise down their impressions of an agent’s intentions than to revise them upwards. Fav=Unfav entails that Favorable and Unfavorable external states have equal and opposite effects. Unfav>Fav entails an expectation that Unfavorable external states can worsen behavior (e.g. excuses), while Favorable external states have no effect (i.e. no ulterior motives); here subjects are faster to revise up their impressions of an agent’s intentions than to revise them downwards.

Supplementary Table 1. Model Comparison

| **Model** | | **Fixed Parameters** | **Free Parameters** | **Mean LLR vs Random Model** | **Mean BIC** |
| --- | --- | --- | --- | --- | --- |
| ***Split-HMM*** | |  |  |  |  |
| 1 | *Integrated* dispositional prior only | $\pi_{o2}=0.001$  $\psi_{Split}=0$  $\psi_{Bad}=0$ | ${1<\pi}_{o1}<5$  $0.1<\pi_{s1}<3$  ${0<\psi}_{Ext}<1$ | 5.39 | 162.9 |
| 2 | *Bad* and *Good*  dispositional priors only | $\pi_{o2}=0.001$  $\psi_{Split}=1$ | ${1<\pi}_{o1}<5$  $0<\psi_{Ext}<1$  $0<\psi_{Bad}<1$ | -53.2 | 280.1 |
| 3 | *Bad*, *Integrated* and *Good* dispositional priors | $\pi_{o2}=0.001$ | ${1<\pi}_{o1}<5$  $0.1<\pi_{s1}<3$  $0<\psi_{Ext}<1$  $0<\psi_{Bad}<1$  $0<\psi_{Split}<1$ | 17.9* | 144.9^**^ |
| ***HGF*** | |  |  |  |  |
| 4 | Two-level HGF | None | $\omega_{Bad}$  $\omega_{Good}$  $\varepsilon_{Bad}>0$  $\varepsilon_{Good}>0$ | 9.83 | 157.5 |

*Note.* Split-HMM: Split Hidden Markov Model; HGF: Hierarchical Gaussian Filter; Mean LLR: mean log likelihood ratio across participants; Mean BIC: mean Bayesian Information Criterion across participants. *Likelihood ratio test vs Model 1: mean LLR = 12.5$\chi^{2}(2)=25.0$ p<0.0001. **Best fitting model by BIC.
